# Transforming women’s and providers’ experience of care for improved outcomes: A theory of change for group antenatal care in Kenya and Nigeria

**DOI:** 10.1371/journal.pone.0265174

**Published:** 2022-05-03

**Authors:** Lindsay Grenier, Brenda Onguti, Lillian J. Whiting-Collins, Eunice Omanga, Stephanie Suhowatsky, Peter J. Winch

**Affiliations:** 1 Maternal and Newborn Health Unit, Jhpiego, Baltimore, Maryland, United States of America; 2 Global Programs, Jhpiego, Nairobi, Kenya; 3 Department of Population, Family, and Reproductive Health, Johns Hopkins Bloomberg School of Public Health, Baltimore, Maryland, United States of America; 4 Department of Monitoring, Evaluation, and Research, Jhpiego, Nairobi, Kenya; 5 Department of International Health, Johns Hopkins Bloomberg School of Public Health, Baltimore, Maryland, United States of America; National Institute of Public Health, MEXICO

## Abstract

**Background:**

Group antenatal care (G-ANC) is a promising model for improving quality of maternal care and outcomes in low- and middle-income countries (LMICs) but little has been published examining the mechanisms by which it may contribute to those improvements. Substantial interplay can be expected between pregnant women and providers’ respective experiences of care, but most studies report findings separately. This study explores the experience and effects of G-ANC on both women and providers to inform an integrated theory of change for G-ANC in LMICs.

**Methods:**

This paper reports on multiple secondary outcomes from a pragmatic cluster randomized controlled trial of group antenatal care in Kenya and Nigeria conducted from October 2016—November 2018 including 20 clusters per country. We collected qualitative data from providers and women providing or receiving group antenatal care via focus group discussions (19 with women; 4 with providers) and semi-structured interviews (42 with women; 4 with providers). Quantitative data were collected via surveys administered to 1) providers in the intervention arm at enrollment and after facilitating 4 cohorts and 2) women in both study arms at enrollment; 3–6 weeks postpartum; and 1 year postpartum. Through an iterative approach with framework analysis, we explored the interactions of voiced experience and perceived effects of care and placed them relationally within a theory of change. Selected variables from baseline and final surveys were analyzed to examine applicability of the theory to all study participants.

**Results:**

Findings support seven inter-related themes. Three themes relate to the shared experience of care of women and providers: forming supportive relationships and open communication; becoming empowered partners in learning and care; and providing and receiving meaningful clinical services and information. Four themes relate to effects of that experience, which are not universally shared: self-reinforcing cycles of more and better care; linked improvements in health knowledge, confidence, and healthy behaviors; improved communication, support, and care beyond G-ANC meetings; and motivation to continue providing G-ANC. Together these themes map to a theory of change which centers the shared experience of care for women and providers among multiple pathways to improved outcomes.

**Discussion:**

The reported experience and effects of G-ANC on women and providers are consistent with other studies in LMICs. This study is novel because it uses the themes to present a theory of change for G-ANC in low-resource settings. It is useful for G-ANC implementation to inform model development, test adaptations, and continue exploring mechanisms of action in future research.

## Introduction

Over the last five years, there has been concomitant increasing interest in improving the experience of maternal care [1–3] and expanding use of group antenatal care (G-ANC) in low- and middle-income countries (LMICs) [4–9]. G-ANC is an alternative service delivery model to standard, individual care where women of similar gestational age attend ANC together for clinical assessment and care, participatory facilitated learning, and peer support [10, 11].

In 2015 the World Health Organization (WHO) released their quality-of-care framework for maternal and newborn health, giving equal weight to the provision and experience of care, underpinning both with the need for competent and motivated human resources and essential physical resources [1]. Following a scoping review to identify what women want, value, and need in pregnancy [2], WHO then explicitly framed their 2016 ANC guidelines around creating a positive pregnancy experience and recommended G-ANC in the context of rigorous research [3]. This new emphasis on experience of care reflects an evolving recognition of its integral role in service utilization and health outcomes [12]. Less focus has been directed at the provider’s experience of care, which impacts that of their patients via motivation and ability to provide quality respectful care. [13–15]. Although substantial interplay can be expected between pregnant women and providers’ respective experiences of care, findings have generally been reported separately.

For pregnant women, studies of G-ANC in LMICs provide evidence for increased satisfaction [16–20] service utilization, [16, 19, 20–24] health literacy, [16, 17–19, 23] pregnancy related empowerment, [16, 25] uptake of healthy behaviors, [16, 21–24] and improved quality of care [16, 20, 21, 23, 24, 26, 27] compared to individual ANC. Qualitative research related to the experience of women has universally highlighted a preference for G-ANC over individual care and an appreciation for perceived gains in health literacy and meaningful relationships with providers and other women [16, 18, 28, 29].

A systematic review by Lazar et. al. of providers’ experiences from high- middle- and low-income countries identified three major themes: Giving women the care providers feel they want and need; building skills and relationships; and the value proposition of group antenatal care [30]. The review notes that despite substantial heterogeneity across studies, there was notable concordance of experience, one that was overall positive despite challenges caused by organizational barriers [30].

Where similar constructs or outcomes have been examined for women and providers, substantial concordance has been reported, both in parallel personal experience (e.g., strengthened patient-provider relationships; satisfaction; improved quality of care) and provider perceptions of women’s experience (e.g., increased comfort communicating with provider) [28–36]. This concordance is exemplified by two phenomenological studies in Canada, which found the core meaning of G-ANC for women to be “getting more care than they realized they needed” and for providers to be “providing richer care” [35, 36].

Despite the growing evidence base for the benefits of G-ANC in LMICs, its theoretical grounding remains underdeveloped, and the mechanisms linking provider experience to efficacy undefined [30, 37]. We were only able to identify one previously published theory of change (TOC), which omitted providers and lacked clear documentation of an empirical evidence base [18]. The objective of this study is to explore the experience and effects of G-ANC on both women and providers to inform an empirically based integrated TOC to aid future research and implementation efforts.

## Methods

This paper reports on multiple outcomes from Jhpiego’s pragmatic cluster randomized controlled group care trial conducted in Kenya and Nigeria as they relate to our study objective (Table 1). The trial applied qualitative and quantitative methods to examine care from the first ANC visit through one-year post-partum, including four “Healthy Mother Healthy Baby” postpartum meetings. This paper presents analysis of G-ANC findings only. Detailed study methodology, ethical considerations, participant characteristics, and primary outcomes of the parent trial are reported elsewhere [6, 21].

**Table 1:** Overview of data sources and corresponding objectives.

Type	Objectives of data collection	Participants
**QUALITATIVE DATA**		
**Focus groups with postpartum women**	To explore women’s experience with participation in G-ANC groups To document perceived benefits and disadvantages of group care	Women enrolled in the study, from one of the last two cohorts, who attended at least one G-ANC meeting. Women were purposefully sampled to include representation from each level of health facility; urban and rural; various ethnic and linguistic groups; adolescents; and primiparous and multiparous women.
**Focus groups with providers at conclusion of last G-ANC meetings**	To explore providers’ experience with delivery of group care To document perceived benefits and disadvantages of group care To document perceived benefits and disadvantages of group care	All providers enrolled in the study who were trained to facilitate G-ANC were invited to participate.
**Individual interviews with postpartum women**	To further explore ideas, experiences, and preferences mentioned in focus groups To examine reasons for low attendance at G-ANC meetings To elicit perceived impact of G-ANC on women experiencing complications during pregnancy	Purposefully sampled women enrolled in the intervention arm who: only attended 1–2 G-ANC meetings; attended 4–5 meetings but still delivered at home; experienced a complication during pregnancy; or exhibited high participation in FGD. Not all women interviewed participated in an FGD.
**Individual interviews with providers at conclusion of last G-ANC meetings**	To further explore ideas, experiences, and preferences documented in focus groups	A subset of providers participating in focus groups who met one of the following criteria: Worked at a high or low ANC census facility; exhibited high or low enthusiasm and participation during FGD
**QUANTITATIVE DATA**		
**Survey of pregnant women at enrollment**	To examine effects of G-ANC on: Perceived quality of care† Satisfaction with care Utilization of services† Confidence in ability to communicate, make decisions, and take action related to health Knowledge and uptake of health-promoting behaviors Preferences for model of care Communication with providers outside of ANC; communication with G-ANC group outside of meetings*	All women enrolled in the study, including intervention and control arms *Communication with other women only asked in the intervention arm
**Survey of women 3–6 weeks post-partum**		
**Survey of women 1-year post-partum**		
**Survey of providers at enrollment**	To examine effects of GANC, compared to individual care, on: Perceived quality of care provided to women Satisfaction with providing care to women Preference of group or individual care	Providers chosen to facilitate G-ANC and enrolled in the study: 3 per facility in intervention sites
**Survey of providers upon completion of all G-ANC cohorts**		

### Study design and setting

The trial was conducted from October 2016–November 2018 in Nasarawa State, Nigeria, and Kisumu and Machakos counties in Kenya. All study areas have decentralized health systems. Compared to Nigeria, Kenya has higher female literacy (78% versus 53%); contraceptive prevalence (61% versus 17% among women aged 15–49); proportion of pregnant women receiving ANC (94% versus 67%); and births assisted by skilled attendants (62% versus 43%) [38]. Each study area includes rural agricultural land and peri-urban areas, with Kisumu bordering Lake Victoria; Machakos, Nairobi; and Nasarawa, Abuja. In collaboration with local health authorities, 20 health facilities with a minimum of two ANC providers on duty at a time were selected in each country, pair-matched and then randomized to either continue offering standard individual ANC or introduce G-ANC. Urban, peri-urban, and rural facilities ranging from health centers to hospitals were matched and included in each country. Detailed site selection and matching criteria are reported by Kabue et al. [6]. Both service delivery models followed Kenyan and Nigerian clinical protocols, respectively. All intervention sites continued offering individual ANC to women not enrolled in the study and as needed or requested by those participating in G-ANC. The study was designed in consultation with the Nasarawa State Ministry of Health (MOH), Nasarawa Primary Health Care Development Agency, the Kenyan National MOH, and health departments in Kisumu and Machakos Counties. Ethical approval for the study was obtained from Nasarawa State MOH, KEMRI SERU, and Johns Hopkins Bloomberg School of Public Health Institutional Review Board. This trial is registered with Pan African Clinical Trials Registry (PACTR201706002254227), the study protocol and quantitative study tools are published [6], and the quantitative data is publicly available (DOI: 10.6084/m9.figshare.9744899).

### Group ANC model, meeting framework, and implementation

**Fig 1:**
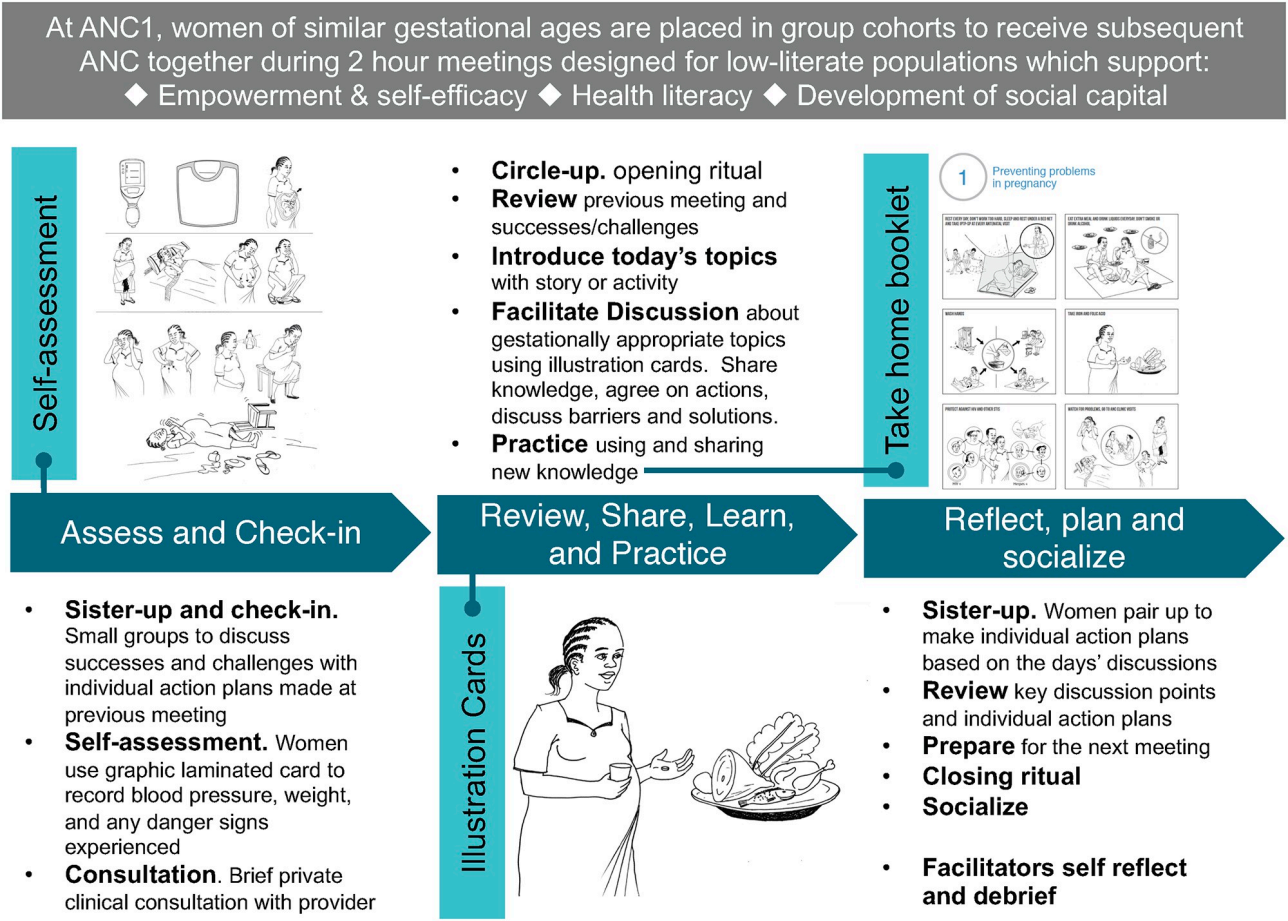
Group antenatal care (G-ANC) meeting framework. Images republished from Jhpiego’s 2016 five-meeting Group Antenatal Care package under a CC BY license, with permission from Jhpiego corporation, original copyright 2016.

The meeting framework developed for the study was informed by previous G-ANC models [39], American College of Nurse-Midwives Home Based Life-Saving Skills methodology [40], and principles for best practices developed by the Global G-ANC Collaborative [1]. During women’s first (individual) ANC visit, they were invited to join a G-ANC cohort for subsequent routine care. Cohorts consisted of 8–15 women of similar gestational age, and visits followed a pre-determined schedule, including five 2-hour long meetings facilitated by ANC providers. Meeting activities were designed to decrease hierarchy and increase participation in care; identify and address barriers by promoting peer-to-peer learning and problem solving; build group cohesion and social support; and empower and nurture self-efficacy and action planning for specific behaviors. Each of the five meetings addressed gestationally appropriate topics and followed the same framework: assess and check-in; review, share, learn, and practice; and reflect, plan, and socialize (Fig 1). (See S1 Appendix for additional information on meeting materials).

Providers facilitating G-ANC participated in an initial weeklong training followed by ongoing onsite mentoring and opportunities to meet with facilitators from other facilities to share successful strategies and problem solve together. Efforts to maintain model fidelity included self-reflective quality assurance and improvement tools and structured post-meeting debriefs (See S2 Appendix for related tools). Additional implementation details can be found in prior publications [6, 21].

### Participant recruitment and data collection

Participants consented to longitudinal data collection at the time of enrollment. Only those in the intervention arm were consented to potential participation in focus groups and interviews. Data collection was conducted in English, Kiswahili, Kamba and Dholuo (Luo) Kenya, and English and Hausa in Nigeria, by native speakers.

#### Surveys

Women ≤24 weeks gestational age (GA) attending their first ANC visit were screened for eligibility, consented, and enrolled into the study. Additional eligibility criteria included minimum age of 15 years; ability to provide locator information; and plans to stay in the area for the following year. Surveys were administered to all enrolled women in the health facility at enrollment and in participant’s homes postpartum (3–6 weeks and one year). Three ANC providers per intervention site were chosen by site administration staff and invited to be trained as G-ANC facilitators. Participating providers completed surveys before and after completing all ANC meetings for study cohorts (i.e., minimum of four). All survey data were directly entered into REDCap^™^ on tablets by trained research assistants.

#### Focus groups and interviews

Focus group discussions (FGDs) and in-depth semi-structured interviews were conducted with G-ANC providers and a sub-set of recently delivered women enrolled in the intervention arm after cohorts finished their last G-ANC meeting. A purposeful sample of women who had participated in one of the last two cohorts and attended at least one meeting were invited to participate in focus groups specific to primiparous women; multiparous women; or adolescents. Further sampling strategies were employed to ensure representation from each level of health facility, urban and rural locations, and various ethnic and linguistic groups. A total of eighteen focus groups were planned to meet this criteria with ten to fifteen women invited to each. All G-ANC providers were invited to one of four provider-specific FGDs. Interviews were planned for deeper exploration of specific topics, without a priori sampling targets (See Table 1 for details). Separate but similar facilitation guides were used across FGDs and interviews which probed the experience of providing and receiving G-ANC, perceived effects, and recommendations for future implementation (S3 Appendix). All participarts were reimbursed for transportation, and no other incentive was provided. Experienced qualitative researchers who spoke local languages and were not otherwise involved in study implementation were trained and initially supervised by author PW to conduct FGDs and interviews using semi-structured guides alongside study research assistants trained to act as notetakers during FGD sessions. Debriefs were conducted at the end of each day to draft cover sheets summarizing key themes for each session. PW also participated in FGDs, and interviews conducted in English or Kiswahili. All FGDs and interviews were recorded and later transcribed and translated by native speakers present at the time of recording.

### Analysis and development of TOC

#### Qualitative analysis

We adopted a multi-phased iterative approach to framework analysis to develop an empirically based theory of change for G-ANC. Qualitative data were managed and analyzed using both ATLAS.ti and Excel software. Phase 1) Familiarization: Initial coding was completed by BO, EO, and LC utilizing broad descriptive codes generated by review of three transcripts by each researcher and discussed in a four way conversation with PW. The full study team then identified the subset of descriptive codes related to experience and effects of G-ANC. Phase 2) Theme identification: Examining the subset of data within those codes, LG then used semi-directed content analysis informed by field observations, published literature, and discussions within the Global G-ANC Collaboration, to generate an expansive set of codes potentially related to a TOC based on open, in vivo coding. 3) Indexing: Codes were reviewed and refined with the larger research team before returning to the raw transcript data to recode using the new schema. 4) Charting: The resulting subset of data was organized within a matrix to visualize the relevance of codes across countries, providers, and recently delivered women. Codes were grouped into larger categories at this stage. 5) Interpretation/Mapping: diagram drawing was used to further refine and collapse subthemes within broader categories and place relational concepts within a TOC.

#### Quantitative analysis

Next, as a validity check, the quantitative data were reviewed for content relevant to the individual concepts included in the TOC, and findings were compared (Table 1). We applied generalized estimating equations with generalized linear models clustering at the facility level to assess women’s perceptions of quality, satisfaction, and ANC model preference; health knowledge and uptake of health promoting behaviors; and communication with providers outside scheduled ANC by study arm. Bivariate difference in difference analysis was employed to assess comparative changes from enrollment to 3–6 weeks postpartum between study arms for women’s confidence in communication, skillful decision making, and locus of control related to maternal, newborn, and reproductive health. Descriptive statistics were performed for provider data (due to small sample size) and data collected only in the intervention arm (due to lack of comparator). Analyses were completed in R statistical software (R Core Team, 2017) and Stata15.

## Results

### Participant characteristics

The study enrolled 2,088 women from October 2016 to June 2017, with even distribution across 20 clusters in each country. Demographic details of enrolled women and trial profiles for each country have been previously published [21]. A total of 88.3% (1844/2088) of enrolled women completed the 3–6 weeks postpartum survey, and 72.0% (1504/2088) completed the one-year postpartum survey. Seventy-five ANC providers were enrolled and trained as G-ANC facilitators, including midwives, nurses, and community health extension workers. (See S1 Table for additional details). We conducted 19 FGDs with women and 4 with providers (averaging 120 minutes); and 42 interviews with women and 4 with providers (ranging from 6–122 minutes) (Table 2).

**Table 2:** Qualitative and quantitative data collection by country.

	Total N(%) [# of participants]	Nigeria N(%) [# of participants]	Kenya N(%) [# of participants]
**Women**	**N = 2088**	**N = 1075**	**N = 1013**
**Focus group discussions**	19 [177]	8 [91]	11 [86]
**Interviews** *	42	13	29
**Survey**:			
**At enrollment**	2088 (100)	1075 (100)	1013 (100)
**3–6 weeks post-partum**	1844 (88.3)	1018 (94.7)	826 (81.5)
**1-year post-partum**	1504 (72.0)	873(81.2)	631(62.2)
**ANC Providers**	**N = 55**	**N = 30**	**N = 25**
**Focus group discussions**	4 [41]	2 [20]	2 [21]
**Interviews** *	4	3	1
**Survey**:			
**At enrollment**	54 (98.2)	29 (96.7)	25 (100)
**After completion of all G-ANC study cohorts**	55(100)	30 (100)	25 (100)

*All interviewed providers participated in FGDs; most, but not all women interviewed also participated in an FGD.

### Themes

Seven inter-related themes arose representing the experience and perceived effects of group ANC in contrast to individual care. Three themes relate to the shared experience of care of women and providers, and four relate to effects of that experience, which are not universally shared (Table 3). Supporting data was provided by both women and providers for all themes (i.e., providers validated data relating to women’s experience and vice versa).

**Table 3:** Themes related to the experience and effects of G-ANC by women and providers.

Themes	Sub-themes	Experienced By	
		Women	Providers
**EXPERIENCE OF G-ANC: personally fulfilling relation-based responsive and empowering care**			
Forming supportive relationships and open communication	“One-ness”: Feeling part of a family, loved, cared for, and respected	X	X
	“Being free”: Ability and courage to ask questions, share ideas and make mistakes	X	-
Becoming empowered partners in learning and care	Shared workload and engagement in care	X	X
	Collective teaching, learning, and problem solving	X	X
Providing and receiving meaningful clinical services and information	“More than clearing the queue”: Provision and receipt of comprehensive, responsive care	X	X
	Understanding health status and care	X	-
	“Once and well”: Deep topic exploration with actionable information shared and understood	X	X
**EFFECTS OF G-ANC EXPERIENCE: opportunities for improved Outcomes**			
Self-reinforcing cycles of more and better care	Improved quality of care	X	X
	Increased patient and provider satisfaction	X	X
	Improved motivation and ability to pursue follow-up for routine care and complications	X	X
Linked improvements in health knowledge, confidence, and healthy behaviors	Gaining knowledge, confidence, and courage to take specific actions	X	-
	Pride in new knowledge, skill, and ability to act	X	-
	Ripple effect beyond G-ANC members	-	-
Improved communication, support, and care beyond G-ANC meetings	Increased social capital and access to peer and provider support outside of meetings	X	-
	Improved uptake of non-G-ANC clinical services, including facility-based delivery and post-partum care	X	-
	Improved experience of care beyond G-ANC meetings	X	-
Motivation to continue providing G-ANC	Reduced fatigue and stress	-	X
	Increased personal and professional fulfillment	-	X

#### Experience of G-ANC: Forming supportive relationships and open communication

Supportive relationships among and between women and providers in G-ANC emerged as highly valued by both parties and a defining characteristic distinguishing G-ANC from individual ANC. These relationships, in turn, provided the scaffolding for improved communication.

Women reported feeling supported, loved, cared for, respected, and unjudged in G-ANC. As one woman described, “*they really cared and were patient with me…they were making way for me…I felt I was in a home*, *I was loved” (Woman*, *NG)*. In both countries, women used the term “one-ness” to define the sense of community they felt within their group, where *“we love each other*” *(Woman*, *KE)* and “*live as friends*, *as if we come from one family*” *(Woman*, *KE)*. As one woman explained:

*The service providers were approachable*, *if you have a problem*, *they could be free to help you*, *they never judged anybody*, *they could just be with us as fellow mothers*. *They were free to laugh with us…they could dance with us*, *laugh with us*.

*(Woman*, *KE)*

In contrast, women expressed that individual ANC was a place where “*there is no one-ness like this one*” *(Woman*, *NG)*, “*you feel lonely*” *(Woman*, *KE)*, and fear “*they [providers] will not keep your secrets*” *(Woman*, *NG)*.

The mutual trust created by this experience of “oneness” provided the foundation for “being free” within the group. Women described having the ability and courage to ask questions, share problems and ideas, and make mistakes:

*Being in the group we had time to interact with [service providers]…You understand they are not bad people*, *when you tell them your problems they will care*, *so as we understood them*, *we opened up*. *Because when in a group there is that freedom…[In individual ANC]…you look at [the service provider]*, *you fear even the looks*, *how will I tell her my problem*? *But when in a group you understand each other*, *you laugh together*, *you sit together*, *you are free*.

*(Woman*, *KE)*

Provider’s echo these feelings, taking enjoyment from the closeness among women, and their own improved relationships and communication with their clients.

*With group ANC I have seen a very improved relationship*, *client health worker relationship*. *As interactive as it is we have been able to bond*. *You know with the individual one is like we acted the boss…but now we interact at the same level*, *we bond with each other*, *we talk on phone*, *they consult anytime of the day*, *and by the end of the day we find some kind of job satisfaction as health workers*

*(Provider*, *KE)*

*What I enjoyed most is the relationship between the care giver and the client*. *At some point you will get the client calling you by name*, *she forgets that you are the sister then she starts telling you “[provider’s first name]*, *you know this this this and that” and then you will also know them by name*

*(Provider*, *KE)*.

Women and providers linked the interactive nature of G-ANC meetings, decreased hierarchy, community forming activities, collective norm-setting (e.g., for confidentiality and respect of all opinions), continuity of care, and increased touch time to the development of supportive relationships, mutual trust, and open communication.

#### Experience of G-ANC: Becoming empowered partners in learning and care

Providers and women describe a shift from viewing the provider as the sole source of information and care in individual ANC to viewing these as shared responsibilities in G-ANC. This new partnership was characterized by a shared workload (e.g., self-assessments); collective problem solving where, “*everyone is embracing each and everyone’s problem*” *(Provider*, *KE)*; and an understanding that everyone is a teacher, and everyone is a learner. Both parties valued this new partnership and voiced advantages for themselves and others.

Women felt pride in their ability to assess themselves and each other, highlighting “*The difference is*, *the things they will do for you in individual ANC*, *in group ANC we will do it for ourselves*” (Woman, NG). Providers note how this contributes to increased partnership in their own care. As one gives by way of example, “*When they discover that their weight dropped*, *they themselves*, *they will notice and they will call our attention “ah*, *ah*, *Aunty Nurse*, *why is it that my weight dropped today*?” *(Provider*, *NG)*.

Multidirectional collective problem-solving and learning was described—between women, between women and providers, and between providers:

*We would share experiences*. *Someone would say that which she has undergone*, *another person also says*, *and then we see how we could help*. *We were working together*. *Everything we were doing we were coordinating*. *For example*, *like measuring weight*, *[or] teaching each other…Like*, *there was a day someone asked a question concerning whether someone can have sex during pregnancy*. *Someone said “no”*, *others*, *“It is normal you can just have sex”*, *Another said*, *“No you shouldn’t have sex until after delivery*.*” Then we later found a solution*

*(Woman*, *KE)*.

Providers found this collective learning and problem solving not only beneficial for the women, but for themselves as well:

*The clients are empowered*…*in normal ANC we assume that the clients do not know*. *But we realize that they know so much more than even what we know*, *so when we empower them and allow them to give us their ideas first then talk about ours*, *they get so much help—both the clients and ourselves*

*(Provider*, *KE)*

Self-assessments, actively seeking knowledge and ideas from women (based on the structured discussion methodology), and components which decrease hierarchy (such as sitting in a circle) were viewed as aspects of G-ANC which contributed to these new partnerships. Women and providers also described a feedback loop between feeling like partners in care and their improved relationships with each other.

#### Experience of G-ANC: Receiving and providing meaningful clinical services and information

The experience of G-ANC was most succinctly summarized by women and providers as they contrasted it to individual ANC, which both parties characterized as rushed, incomplete, impersonal care focused on “clearing the queue” (aka “finishing” the waiting line of women). In contrast, G-ANC was credited with providing a supportive environment capable of responding to women’s needs.

Women cited three key advantages of G-ANC compared to the “clearing the queue” model of individual ANC: 1) being asked about their problems and given the opportunity to ask questions; 2) having their health status and/or treatment explained to them; and 3) being taught how to care for their and their baby’s health.

*There is a difference between this and the general antenatal*, *because here we discuss in detail*, *we ask ourselves questions*, *we are given manuals and we discuss as one family*, *but it is not so with the general one because people are too much*, *you are not opportune to ask questions*, *also we don’t discuss in detail*.

*(Woman*, *NG)*

*[In response to what care they would recommend to a friend] I would like to tell her to come to the group where there are many teachings*, *but in the other one they just examine you and you are not taught*. *Even if something is happening to you*, *you don’t know*

*(Woman*, *KE)*

Providers highlighted how G-ANC helped them to “*get out of poor-quality clearing and forwarding routine ANC*” *(Provider*, *KE)* and “*provide holistic care to mothers*” *(Provider*, *KE)*, in part through feeling more invested in the care they were providing:

*[In individual ANC] you want to finish your work; you just do it quickly*. *You finish your work and forget about them*. *But here [G-ANC] you are*—*you want to finish*, *you want to talk to them*, *you want to assess them*, *you want to interact with them*, *so it is a different perception*.

*(Provider*, *KE)*

Providers outlined a number of specific ways G-ANC supported provision of meaningful care: scheduled meetings allowed anticipatory staffing and relieved provider pressure to “clear the queue”; the structured materials, methodology, and increased touch time allowed them to cover topics deeply while responding to women’s interests and concerns; and strengthened provider-patient relationships meant women were more likely to share specific concerns and providers were more motivated to respond with individualized care.

#### Effect of G-ANC experience: Self-reinforcing cycles of and more and better care

Qualitative data indicates that the experience of care in G-ANC contributed to mutual satisfaction and a self-re-enforcing cycle of more and better care. This cycle was characterized by higher rates of satisfaction, preference for the G-ANC model in future care, and perceptions of improved quality of care, utilization of care and follow-up. Quantitative data support these qualitative findings.

Women credited both the value and the enjoyment they derived from G-ANC with an increased desire to return for additional ANC. As a woman who was initially skeptical of G-ANC explained:

*When I came for the first time*, *I really enjoyed it*. *In fact*, *I felt as if we should not close for the day*. *When I went back home that day*, *I told my husband that I don’t mind we should be going for group ante-natal every day…[it] is very special*. *There you meet new people and learn a lot from them too*.

*(Woman*, *NG)*

Women further highlighted how their experience of care improved their ability to access appropriate care. For example, one woman described how in past pregnancies she had been too scared to explain her problems to her provider and had suffered multiple losses. After gaining an understanding of the importance of her symptoms (through collective learning) and having the comfort and courage to discuss with her provider (through forming supportive relationships, and becoming an empowered partner in care), she was able to get the additional care she needed to prevent another loss.

Women in G-ANC were more likely than women in individual ANC to agree with statements of quality care and to report being “very satisfied” with the ANC received. Over 95% would prefer it over individual care in a subsequent pregnancy (Table 4).

**Table 4:** G-ANC effect on client perceptions of quality, satisfaction, and ANC model preference.

	Nigeria			Kenya		
	Intervention (n = 510) N(%)	Control (n = 508) N(%)	p-value	Intervention (n = 415) N(%)	Control (n = 411) N(%)	p-value
**PERCEPTIONS OF QUALITY**						
**I had enough time with my provider**.	491 (96.3)	444 (87.4)	0.012	406 (97.8)	360 (87.6)	<0.001
**I was able to ask questions and they were answered in a way I understood**.	492 (96.5)	419 (82.5)	0.001	401 (96.6)	380 (92.5)	0.050
**I learned important things about how to keep myself and my baby healthy**.	500 (98.0)	425 (83.7)	<0.001	402 (96.9)	354 (86.1)	0.004
**MEASURES OF SATISFACTION**						
**During my next pregnancy, I would be happy to receive care similar to what I received during my last pregnancy**.	490 (96.1)	448 (88.2)	0.009	405 (97.6)	360 (87.6)	<0.001
**I am very satisfied with the antenatal care I received**.	489 (95.9)	430 (84.6)	0.002	407 (98.1)	346 (84.2)	<0.001
**Preferred model for receiving ANC** *						
Group-based care	487 (95.5)	236 (46.5)	NA	398 (95.9)	236 (57.4)	NA
Individual care	23 (4.5)	272 (53.4)	NA	236 (4.1)	175 (42.6)	NA

Subjects were asked if they agreed, disagreed, or neither agreed nor disagreed with each statement in relation to ANC they received during their last pregnancy at 3–6 weeks postpartum; agreed is shown.

*a brief description of G-ANC was read to women in the control group as part of the question.

Providers universally felt they were able to provide higher quality care through G-ANC, highlighting their improved ability and motivation to 1) provide in-depth consistent health education; 2)identify and manage complications; and 3) provide “consistent” and “comprehensive” follow-up. They cited the structured meeting materials and decreased need for repetition, improved relationships, open communication with women, continuity of care, and G-ANC cohort specific registers as key drivers:

*In group ANC the staffs are focused*, *the clients are focused*. *So*, *we all know what to expect for every meeting*. *To me it saves time*, *and you give quality*, *just like [R8*, *R3*, *R2] have said*. *You give quality because*, *ahhh*, *if I start talking about what to expect—nutrition*, *HIV*, *danger signs—to individual clients*, *and I want to see 30*, *by the time I see the sixth I am tired*. *So*, *the rest will just… I will just be clearing them*

*(Provider*, *KE)*

*Early identification of conditions and managing them promptly—we’ve realized this can be achieved so well through group ANC*. *In individual ANC when I see a mother who has come with an STI I tell them to go and get a drug*, *they do not come back*, *I do not even realize that they didn’t come*, *unlike group ANC which now I have these mothers at my fingertips*.

*(Provider*, *KE)*

*In group ANC you will be able to know your target number and if they are not there you call*. *But in other ANC*, *whoever is present*, *you don’t even know who doesn’t come or who comes…With this group ANC*, *it motivates us as a duty to do*, *but in other ANC*, *nobody cares whether they come or they don’t come*, *you don’t really bother to locate them*.

*(Provider*, *NG)*

As compared to individual care, more providers reported feeling they were able to meet their own goals of quality care with G-ANC (Table 5). The number of providers who reported their ANC related job satisfaction as “extremely satisfied” doubled in both countries, and none chose individual care over G-ANC when asked their preferred model (Table 5).

**Table 5:** G-ANC effect on provider perceptions of quality, satisfaction, and ANC model preference.

	Nigeria		Kenya	
	Baseline* N = 29 N(%)	Endline* N = 29 N(%)	Baseline* N = 25 N(%)	Endline* N = 25 N(%)
**PERCEPTIONS OF QUALITY**				
**I am able to provide level of quality in ANC that I would like to provide: Agree**	25 (86.2)	29 (100.0)	21 (84.0)	25 (100.0)
**I have enough time with my patients: Agree**	24 (82.8)	29 (100.0)	17 (68.0)	25 (100.0)
**Sometimes I worry that patients leave their visits with unanswered questions or confused about some things: Agree**	21 (72.4)	0 (0.0)	7 (28.0)	2 (0.0)
**MEASURES OF SATISFACTION**				
**ANC related job satisfaction: extremely satisfied** ^†^	13 (44.8)	25 (86.2)	7 (28.0)	14 (56.0)
**Enjoy providing ANC: A Lot** ^†^	14 (48.3)	26 (89.7)	18 (72.0)	22 (88.0)
**Preferred model for providing ANC**				
Group-based care	-	27 (93.1)	-	24 (96.0)
Individual care	-	0	-	0
Both equally	-	2 (6.9)	-	1 (4.0)

*All providers were providing individual ANC at baseline, and a mix of individual and group ANC at endline. Baseline questions specifically referred to individual ANC and Endline questions specifically to G-ANC.

^†^Asked on a five-point scale, those selecting 5, “extremely satisfied” and enjoy “a lot” shown.

#### Effect of G-ANC experience: Linked improvements in health knowledge, confidence, and healthy behaviors

Women placed great value in the exchange of relevant, actionable information to support healthy pregnancies and the transition to motherhood. They expressed feeling supported and empowered through G-ANC to access information, make decisions for themselves, and take action:

*I feel good because now I can assist another person*, *and if I am pregnant*, *I am able to take action*. *That is a very great achievement and I respect my service provider for empowering me*

*(Woman*, *KE)*

Discussions related to health knowledge and behaviors were often anchored in concrete examples of how women translated knowledge to action in the service of themselves, their children, and their greater communities. Most stories related to 1) improved recognition and response to danger signs; 2) better understanding of and ability to enact health-promoting behaviors; or 3) the ability to advocate for behavior change and care-seeking (see S2 Table for examples of each within and beyond women’s households). Many women reported becoming teachers and sources of health advice in their communities, from which they derived pride, “*It is something that one is proud to have…with that confidence you can help the society*” (Woman, KE).

Providers likewise perceived and valued a shift in understanding and engagement among women in G-ANC as compared to individual care:

*What I enjoyed most about the group is the way the women are understanding what they are being taught and are expected to do*…*they are much committed to what they should do for themselves and are more empowered in participating towards their care*

*(Provider*, *NG)*

Women and providers explicitly linked the changes in health knowledge and behaviors to G-ANC’s structure, content, and experience. They emphasized the importance of women having the ability to ‘be free’ and providers the time to ‘go deep’ alongside the meeting activities which actively encouraged women to learn from each other. Women also highlighted the role of provider attitudes in their ability to learn and trust what providers said: “*they were friendly*, *polite and caring and as such we understand what they teach us easily”(Woman*, *NG)*. Take home booklets provided to G-ANC participants were frequently credited with easing the task of sharing information and changing the perceptions of family and community members.

While qualitative findings consistently linked participation in G-ANC to increased confidence with health-related communication, knowledge, and decision making, quantitative analyses show more variability across these areas (Tables 6 and 7). Quantitative findings align with qualitative findings in indicating that women in G-ANC were more likely than those receiving individual ANC to report uptake of health promoting behaviors. However, the strength of this relationship was inconsistent across behaviors and countries (Table 6).

**Table 6:** Confidence in relation to communication, skillful decision making, and locus of control related to maternal, newborn, and reproductive health.

	Nigeria				Kenya				
Thinking about your next pregnancy…[Agree shown]	Inter-vention (n = 510) N(%)	Control (n = 508) N(%)	DID	p-value based on DID	Inter-vention (n = 415) N(%)	Control (n = 411) N(%)	DID	p-value based on DID	
**COMMUNICATION**									
**If I don’t understand something a provider is telling me I will tell them and ask them to explain a different way**	501 (98.2)	491 (96.7)	2.8	0.310	408 (98.3)	401 (97.6)	-1.2		0.842
**I will talk with my husband/family about how to keep myself and our baby healthy**	457 (89.6)	435 (85.6)	5.8	0.250	384 (92.5)	363 (88.3)	2.3		0.340
**I could ask my husband/partner to use a condom if I wanted him to**	378 (74.3)	279 (54.9)	-4.0	0.930	370 (89.2)	319 (77.6)	10.1		0.052
**SKILLFUL DECISION MAKING**									
**I know how to recognize a problem with my pregnancy**	496 (97.3)	453 (89.2)	21.1	0.002	400 (96.4)	370 (90.0)	6.7		0.061
**I know what actions I will take if I think there is a problem with my pregnancy**	492 (96.5)	463 (91.1)	18	0.025	407 (98.1)	358 (87.1)	7.5		0.061
**I know how to recognize a problem with my newborn**	443 (86.9)	418 (82.2)	20.5	0.054	372 (89.6)	336 (81.8)	16.6		0.005
**I know what actions I will take if I think there is a problem with my newborn**	441 (86.5)	416 (81.9)	22	0.063	380 (91.6)	317 (77.1)	15.1		0.003
**I am good at making decisions related to the health of myself and my family**	460 (90.2)	447 (88.0)	18.6	0.077	405 (97.6)	368 (89.5)	5.6		0.025
**LOCUS OF CONTROL**									
**There are things I can do to help prevent problems and keep myself and my baby healthy**	457 (89.6)	402 (79.1)	17.1	0.003	382 (92.0)	354 (86.1)	4.2		0.063
**I am able to make decisions for myself** *	471 (92.4)	409 (80.5)	16.8	0.053	408 (98.3)	401 (97.6)	-1.6		0.982
**I can have some control over if and when I get pregnant again**^†^ **(asked at endline only)**	480 (94.3)	370 (72.8)	NA	<0.001	386 (93.0)	330 (80.3)	NA		0.005

Endline data shown (3–6 weeks postpartum). Difference in difference (DID) analysis from study enrollment (1^st^ ANC) to endline.

*Prompted with: “Thinking about life generally, do you agree or disagree…”.

^†^Only asked postpartum, no DID available.

**Table 7:** G -ANC effect on health knowledge and uptake of health promoting behaviors.

	Nigeria			Kenya		
	Intervention (n = 510) N(%)	Control (n = 508) N(%)	p-value	Intervention (n = 415) N(%)	Control (n = 411) N(%)	p-value
**HEALTH KNOWLEDGE**						
Able to name ≥5 danger signs of pregnancy	244 (47.8)	153 (30.1)	0.063	177 (42.7)	119 (29.0)	<0.001
**PRENATAL HEALTH PROMOTION**						
Completed 6/6 Birth Planning Actions*	435 (85.3)	244 (48.0)	<0.001	346 (83.4)	250 (60.8)	<0.001
Chose PPFP method prior to delivery	342 (67.1)	167 (32.9)	<0.001	301 (72.5)	170 (41.4)	<0.001
**POSTNATAL HEALTH PROMOTION**						
Slept under LLIN previous night: mother^†^	372 (72.9)	345 (67.9)	0.197	382 (92.0)	363 (88.3)	0.048
Slept under LLIN previous night: infant^†^	422 (82.7)	385 (75.8)	0.023	388 (93.5)	376 (91.5)	0.220
Took IFAS previous day	192 (37.6)	92 (18.1)	0.005	128 (30.8)	52 (12.7)	<0.001
Breastfed within 1 hour of birth	252 (49.4)	204 (40.2)	0.006	290 (69.9)	281 (68.4)	0.900
Ever took postpartum IFAS^Ω^	290/439 (66.1)	174/434 (40.1)	<0.05	201/308 (65.3)	74/272 (27.2)	0.023

*Identified facility where they planned to give birth; planned how to get there; planned who was going to accompany; saved money in case of emergency; decided who could make decisions in case of emergency; prepared a birth kit.

^†^Nigeria data previously reported by Noguchi et al, 2020 [27].

^Ω^Question administered at one-year post-partum.

#### Effect of G-ANC experience: Improved communication, support, and care beyond G-ANC meetings

Women reported communicating with peers and providers and gaining access to additional support outside of G-ANC meetings. Support provided by both peers and providers included advice and emotional support; occasional material support (e.g., food, transport money, pampers); and home visits (e.g., after missing a meeting, having a complication, or delivering). In addition, providers facilitated referrals (particularly helping women find openings for facility delivery during strikes in Kenya) and women experienced prompt respectful clinical care from them outside of meetings. Providers encouraged and enjoyed this additional communication and care.

As one woman explained, “*We had unity*. *When one is in trouble you call a friend*, *who is a group member*” *(Woman*, *KE)*. Women made a point to note that these were new sources of support, explicitly due to the community formed during G-ANC:

*In the group*, *we were so united*, *we loved ourselves*, *we ask after each other*, *we communicate with each other on phone*, *we visit each other*. *In fact*, *even after delivery we care for each other and the babies*. *So*, *they united us like a family even with those we don’t know before*. *We share problems like a family*

*(Woman*, *NG)*

Sharing of personal mobile numbers by providers was not explicitly encouraged through the study but spontaneously arose. As one woman described, “*this time around you don’t need to beg*, *they will be the one trying to pass their number across to you*!*” (Woman*, *NG)*. Providers explained that due to their attachment with women in G-ANC, they were anxious to help and wanted to hear updates. They were also confident that women in G-ANC could differentiate danger signs and would call with more serious concerns than women in individual care.

Quantitative data support these qualitative findings. Eighty-five percent of women communicated with each other outside of group meetings, with a similar percentage believing women from their group would reach out to other members if they had a problem or needed help (S3 Table for topics discussed). Four times as many women in G-ANC called or texted their provider over the course of the study compared to those in individual care (Table 8).

**Table 8:** Communication with providers and other group members outside of ANC.

	Nigeria			Kenya		
	Intervention	Control	p-value	Intervention	Control	p-value
	N = 439	N = 434		N = 316	N = 315	
	N (%)			N (%)		
**Called or text messaged provider during pregnancy**	193 (44.0)	38 (8.8)	<0.001	133 (42.1)	40 (12.7)	<0.001
**Called or text messaged provider after pregnancy**	167 (38.0)	27 (6.2)	<0.001	134 (42.4)	42 (13.3)	<0.001
**OF WOMEN WHO ATTENDED AT LEAST ONE GROUP MEETING**						
	**N = 405** *			**N = 280** *		
**Communicated with women from group outside of meeting times**	355 (87.7)	-	-	229 (81.8)	-	-
**Believes a woman would reach out to other group members if she had a problem/needed help**	351 (86.7)	-	-	222 (79.3)	-	-
**Knows of a specific example**	184 (45.4)	-	-	151 (53.9)	-	-

Questions concerning communication outside of meetings were added to the survey at 1 year postpartum after being identified as a significant phenomenon during qualitative research pertaining to G-ANC meetings.

*Number who attended at least one group meeting.

In contrast with their prior expectations or experiences, women also highlighted attentive, respectful care at their ANC facilities outside of G-ANC meetings. They described prompt attendance by staff if they arrived with a problem and encouraging, respectful care during labor and delivery. This improved care outside of routine ANC was repeatedly cited as a reason they believed other women should attend G-ANC. Providers likewise recognized a shift in the relationship women had with the facilities after attending G-ANC:

*There is that*, *what do I say*—*eeh*, *continuum of care*. *Women came to love the health facilities*. *They bond with the health care providers*, *even when you are walking outside somebody says hi*, *so that fear that when you go to the hospital you will be mistreated or you might be slapped while delivering*, *no one experiences that*.

*(Provider*, *KE)*

#### Effect of G-ANC experience: Motivation to continue providing G-ANC

Providers expressed motivation to continue providing G-ANC as a result of reduced stress and fatigue; belief it better served women and children; and increased personal and professional fulfillment. As one provider replied when asked what she didn’t like about G-ANC:

*I like everything about it*. *(It doesn’t take your time*?*) No*, *it doesn’t take my time*, *and it makes*, *it brings closer relationship between me and the women*. *Whenever I went out*, *like when I go to the market*, *the women will be seeing you*, *calling you*, *this is aunty*, *aunty that use to teach us in group antenatal*. *They respect me so much*. *I feel very happy for that*. *It gives you a sense of*, *like you are really helping*, *as if you’re making a difference*. *That is why I’m very very strong on doing [G-ANC]*. *I don’t feel tired when I have group antenatal*.

*(Provider*, *NG)*

Providers not only appreciated the empowering effect the self-assessments and facilitated discussions had on women, but they also viewed them as helpful in decreasing their own workload. In addition, they reported the structured content of G-ANC reduced their stress and fatigue by simultaneously decreasing the need for repetition and increasing organization and their confidence that all essential topics were being covered:

*I am not exhausted*, *and the women are also not exhausted*, *and the work is done and at the end of the day I have achieved what I wanted because there is meeting one*, *there is meeting two*, *there is meeting three*, *like that*.

*(Provider*, *KE)*

*About the competence*, *you don’t collapse because of burn out…*.*you*. *just go step to step and you handle everything because you have that time*…*and when you handle this mother*, *now you be friendly*, *and now they can feel you and you can feel them*

*(Provider*, *KE)*

Providers linked their increased personal and professional fulfillment to their ability to provide more and better care while forming strong relationships with women in G-ANC:

*I think group ANC has changed my viewing of my work*, *because in individual ANC you usually see that mother and you do not know that mother…But with group ANC they come for subsequent meetings*, *you know the mother*, *even after the mother gives birth you are very happy to know you were attending this mother all through and then she delivered well*. *She has a healthy baby*, *so you feel happier with that work*—*because of you*—*you have seen this mother all through from ANC until delivery*, *and the baby is healthy*

*(Provider*, *KE)*.

### G-ANC theory of change: Transforming the provision and experience of care for improved outcomes

**Fig 2:**
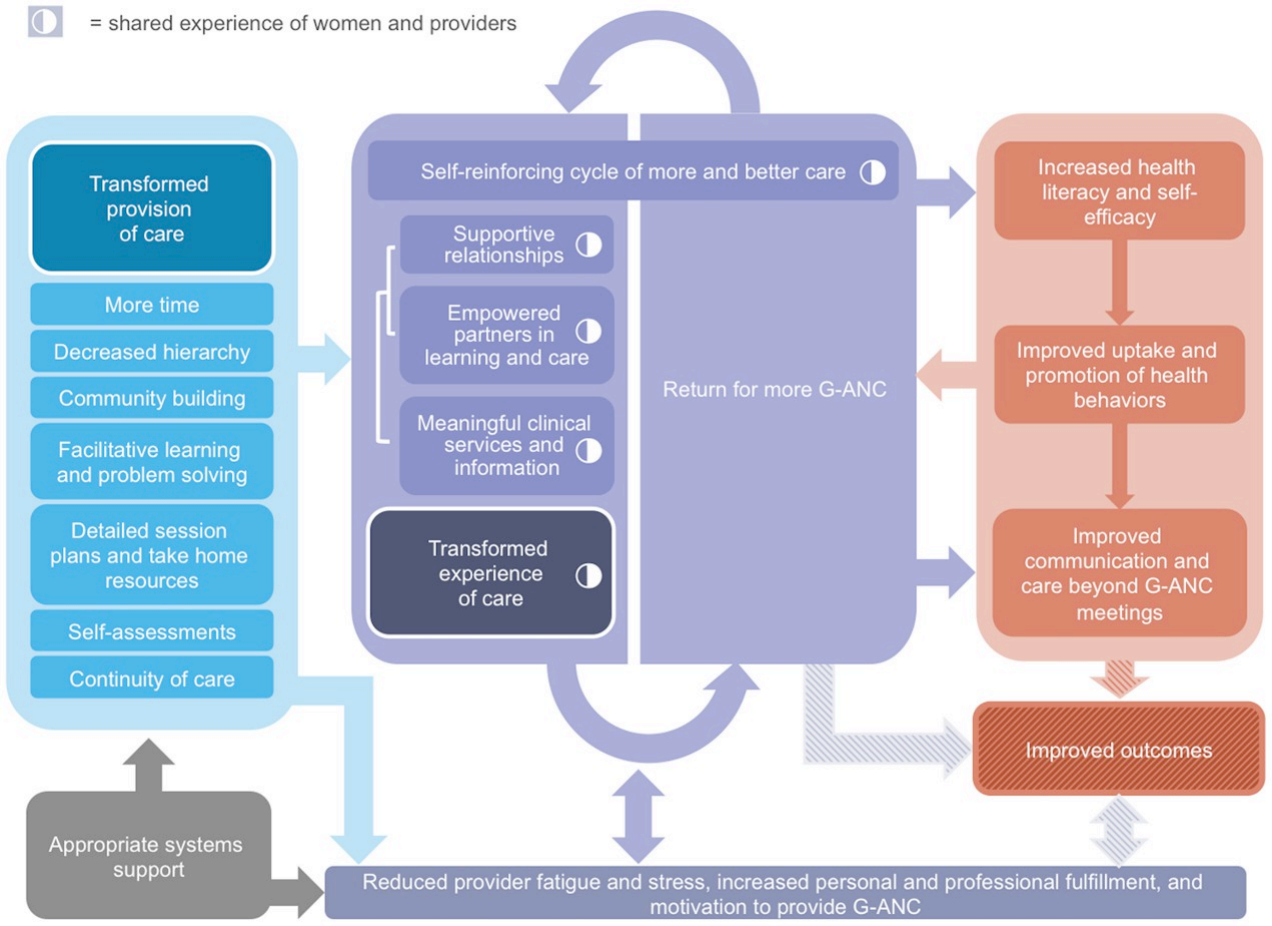
Group antenatal care (G-ANC) theory of change (TOC).

Together these findings support a TOC which centers the experience of care for women and providers on multiple pathways to improved outcomes (Fig 2). Striped arrows indicate the lack of corresponding quantitative data for clinical outcomes.

## Discussion

Our findings support an empirically based TOC which includes the experience and effects of G-ANC on both providers and women. We found the positive experience of G-ANC to be widely shared and the interplay between provider and women’s experience to impact effects on each party.

### Transformed provision of care

The G-ANC model aspects that were referenced as transforming the experience of care included: more touch time; decreased hierarchy; community building (group cohesion activities); facilitative learning and problem-solving; detailed session plans and take-home resources; self-assessments; and continuity of care (Fig 2). Skill practice, individual action planning, and personal/social accountability are incorporated in the model, but did not arise in the qualitative data. One possibility is that these activities may have been cut from meetings due to time constraints, another is that qualitative discussion prompts were not structured in a way to elicit these components as they may relate more to behavior change than experience of care.

### Transformed experience of care

The qualitative analysis revealed a positive feedback loop between forming supportive relationships with open communication and becoming empowered partners in learning and care which reinforces a sense of “one-ness”, or togetherness, among women and providers. This, in addition to the G-ANC meeting framework and content, aides the shift from an experience of providing and receiving care as merely “clearing the queue”, to one of meaningful clinical services and information, culminating in a transformed experience of care for both women and providers. These results are largely consistent with other published qualitative reports on G-ANC. In particular, themes of improved relationships with increased support and open communication are universally reported across studies from both LMICs and high-income countries [16, 18, 30–36].

### Strengthened pathways to improved outcomes

Our results indicate that the experience of G-ANC results in high levels of satisfaction and an expectation of high-value care among women and providers. This creates a self-reinforcing cycle of “more and better care” which increases opportunities for improved clinical outcomes via multiple pathways. The “more and better care” women receive, the more opportunities there are for clinical prevention, identification, and management of problems. In addition, we posit that increasing exposure to G-ANC increases the strength of association between G-ANC and increased health literacy and self-efficacy for specific actions leading to improved uptake and promotion of healthy behaviors, including additional care-seeking (e.g., ANC, facility-based delivery, and postpartum care).

The effects we identified are largely supported by existing quantitative literature on G-ANC in LMICs. Studies have consistently reported higher rates of satisfaction with the model, [16–20] and preference for it over individual ANC [16, 31]. Published reports from nine out of ten LMIC settings, including from this trial, found G-ANC to be associated with increased ANC attendance [16, 19–21, 23, 41, 42]. Likewise, most LMIC based studies, including previously reported findings from this trial, have found some support for improved quality of care [16, 20, 21, 23, 27]; health literacy [16–19, 23]; and uptake of key behaviors [16, 21–24]. Only two previous studies attempt to quantitatively measure G-ANC effect on self-efficacy and/or empowerment, with positive results in Senegal and Malawi but not Tanzania [16, 25]. Similar to results from Senegal [16], we found the most consistent effects for items related to skillful decision making, and little to no effect on measures of communication. This data stands in conflict with our qualitative findings which emphasize changes in patient-provider and familial communication. Similar differences between qualitative and quantitative findings for patient-provider communication were reported in Ghana. [32].

### Provider experience as foundational to change

Our findings establish the shared experience of women and providers in G-ANC and the critical role provider experience plays in the model’s success. Little has been reported specific to providers’ experience of G-ANC in LMICs, and more clarification is needed around the interplay of system support, workload changes, fatigue, and stress. While we found overwhelmingly positive results, in Rwanda, Lundeen et. al. identified “managing altered workloads” as an implementation challenge for G-ANC providers, no differences in a perceived stress scale pre- and post-implementation, and mixed results related to job satisfaction [31]. However, despite those findings, only 5% of providers in their study indicated a preference for individual ANC over G-ANC [31]. A few differences between the two studies may account for the subtle difference in findings. In contrast to Rwanda: 1) the providers in our study had all specifically chosen to work in ANC, had a high degree of control over G-ANC scheduling, and as a rule were not required to cover multiple services during G-ANC meeting times and 2) providers from different facilities were periodically brought together to share lessons learned and collectively problem solve implementation challenges. These differences align with the systematic findings by Lazar et al., including high-income countries, that found “workload was perceived as more onerous in the presence of organizational barriers” but that with proper institutional support, most providers found the benefits outweighed the challenges [30].

### Strengths and limitations

Unlike previously published conceptual models, [18, 43] our revised G-ANC theory of change reflects empirical findings of the experience and perceptions of both providers and pregnant women. It is unique in providing a unified theory of change for G-ANC, presenting themes and pathways conserved across countries and supported by women and providers across a wide range of contexts and participants (e.g., urban, peri-urban, and rural facilities with varying ANC census and levels of care). This study appears to be the first to quantify the effects of G-ANC on communication outside of meetings and to identify improvement in the experience of care beyond G-ANC as a benefit.

Our results could be biased by the original objectives of our G-ANC design process, which have some overlap with TOC components. These validity concerns are mitigated by supportive quantitative data, however, these are only available for themes related to the effects of G-ANC.

In addition, the quantitative portion of the study was not powered to detect differences in clinical outcomes, so we rely on identified associations between G-ANC and commonly used maternal health process indicators to make that link (e.g. ANC4 and facility-based delivery).

Finally, results were obtained under a well-monitored study after limited implementation which may impact generalizability. The design of the study itself ensured that some of the basic institutional support needed for success was provided: facility eligibility required availability of meeting space and more than one ANC provider on duty at a time; all materials required to run sessions were provided; intense mentorship with fidelity checks was provided until providers exhibited ease with meeting mechanics and facilitation skills; and transfers between departments and facilities were limited during the study period. In addition, some findings, such as preferential improved treatment outside of G-ANC, or personal communication with providers outside of meetings (i.e., on their mobiles), might be more prone to change after sustained implementation.

### Recommendations

#### Future research

Future research can explicitly test this TOC to provide a better understanding of the proportional contributions of individual aspects of G-ANC to improved experience and outcomes. We encourage others to retain G-ANC components aimed at skill practice, individual action planning, and personal/social accountability in future G-ANC models and to apply more intentional evaluation to clarify their potential value. We also encourage future research to utilize qualitative data in refining quantitative measurements of empowerment as it specifically relates to self-care and health system interactions during pregnancy. Additional research examining differences between G-ANC and individual-ANC in costing, clinical outcomes, long-term effects on provider experience, and broader health system impacts (including equity) will aid policymakers in prioritizing efforts to improve ANC.

#### Implementation

We encourage those planning G-ANC implementation to give strong consideration to the system supports necessary for success. G-ANC requires a substantial shift in workflow and new skill development for providers. Lazar et al. found providers repeatedly acknowledged anxiety about facilitation skills, and that confidence in these skills went hand in hand with experiencing their groups with satisfaction. In addition, research has shown that model fidelity (inclusive of faciliatory approach) is associated with improved outcomes [44], warranting development of strong mentoring systems and ongoing fidelity checks.

#### Application of findings to individual ANC

While our research is specific to the effects of a group experience, most of the TOC content is not mutually exclusive to individual care. Relationships between provision, experience, and outcomes, may hold across models and are worth exploring. For example, strategies to decrease hierarchy, or perform basic self-assessments (such as the graphical assessment for danger signs) could be applied to individual ANC with relatively minor modifications. “Health talks” commonly given in ANC waiting areas could take lessons learned from G-ANC to effectively integrate more facilitative learning. Likewise, different changes to the provision of individual care could be explored with the objective of creating supportive relationships or partnerships in learning and care.

## Conclusion

We have presented a novel evidence-based theory of change centering the collective experience of care for women and providers as a potent mediator between the provision of care and potential for improved outcomes. We found transformation in provision of care leads to transformation in the experience, expectations, and meaning of care within and beyond ANC meetings creating self-reinforcing cycles of more and better care. The effects of this transformation ultimately reach not only providers and women, but also their families and communities, presenting increased opportunity for improved maternal and newborn outcomes and a positive feedback loop to continue G-ANC. In particular, supportive relationships emerged as central to creating a cascade of inter-related feedback loops spanning the experience and effects of care. This finding reinforces the need to establish trusting supportive relationships as part of quality care in all care settings. All 20 intervention sites from our study continued G-ANC implementation one year after study support ended. A majority continue to provide G-ANC now, despite some disruptions caused by Covid-19. This speaks to the power of supporting positive, fulfilling experiences for providers.

## Supporting information

S1 Appendix Illustrations cards, take home booklet, and material access. Example large illustration card and take home booklet republished from Jhpiego’s 2016 five-meeting Group Antenatal Care package under a CC BY license, with permission from Jhpiego corporation, original copyright 2016.
(PDF)

S2 Appendix Quality assurance tools and mentoring materials: Post G-ANC meeting debrief template, fidelity checklist, and facilitation skills self evaluation. (PDF)

S3 Appendix Qualitative data collection guides. (PDF)

S1 Table Baseline demographic characteristics of enrolled ANC providers at intervention sites. (DOCX)

S2 Table Illustrative quotes discussing G-ANC impact on health knowledge and health promoting behavior within and beyond women’s households. (DOCX)

S3 Table Topics discussed between group members outside of G-ANC meetings. (DOCX)
