# Supplementary material for: Transforming women’s and providers’ experience of care for improved outcomes: A theory of change for group antenatal care in Kenya and Nigeria
Source: PLoS One. 2022 May 3;17(5):e0265174. doi: 10.1371/journal.pone.0265174 (PMC9064109; doi:10.1371/journal.pone.0265174)
Supplement: S1 Appendix — Example large illustration card and take home booklet republished from Jhpiego’s 2016 five-meeting Group Antenatal Care package under a CC BY license, with permission from Jhpiego corporation, original copyright 2016. (PDF) [file pone.0265174.s001.pdf]

Meeting materials

The following materials were created for the study and used by G-ANC providers during meetings:

1. Facilitator's guide
    a. includes an introduction to the study, group care, and facilitation skills; preparation for providing group care; detailed meeting guides; and quality assurance tools
2. A laminated graphic self-assessment card (filled out by women at start of every meeting)
3. G-ANC cohort registers
    a. Includes names & contact info for each cohort member; meeting attendance; and gestationally timed behavioral cues (e.g., asking about specific birth planning components)
4. Large laminated illustrations cards for each meeting topic
5. Printed take home booklets for women to keep

Here we include an example of one illustration card, a job aid which further explains how the cards were used during meetings, and the take home booklet. The illustrations in the take home booklet correspond to the sets of large illustration cards used during each meeting. Please note that the formatting and methodology used with the cards has been updated based on experience gained during the study. To view either the materials used in the study, or the updated materials based on a model for eight ANC visits, please send a request to info@jhpiego.org.

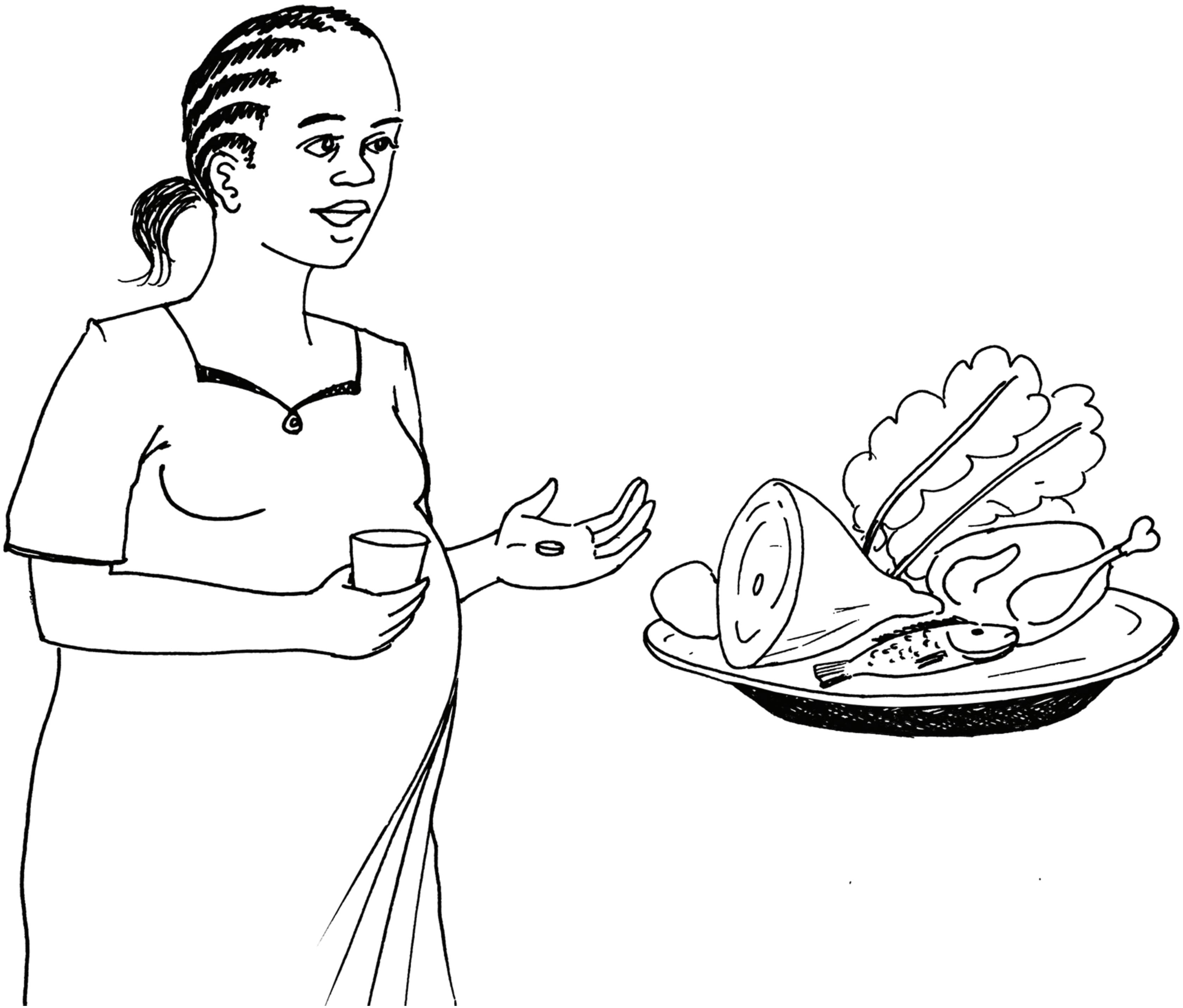

1 Preventing problems in pregnancy

TAKE IRON AND FOLIC ACID

Key Points:

- Iron and folic acid are needed to prevent anemia
- Anemia can prevent your baby from growing well
- Anemia can make you very tired, increase your risk of catching severe malaria and increase your risk of dying in childbirth

To prevent anemia:

- Take an iron and folic acid tablet (blood toner) every day while pregnant and for 40 days after, even if you feel fine
- Get more tablets before you run out
- Eat foods rich in iron and folic acid: red meat, liver, fish, chicken, green leafy vegetables, avocado, sunflower seeds and cooked egg yolk

Facilitator note:

Women often stop taking Iron and folic acid tablets for one of three reasons:

- It makes them constipated (hard to pass stool)
- They run out of tablets
- They start feeling better (after 4-6 weeks)

Make sure that these have been discussed by the end of step 3: Discuss barriers and solutions.

You may also want to ask women for ideas for how to remember to take them everyday.

Problem Cards (2; 5a; 5b)

1. Ask: *What serious health problems have we seen in our community for* ____[state group*: pregnant women/ newborns/ women after birth*]?
    a. Summarize answers
2. Lay down picture cards one by one stating the problem it represents (All CAPS statement on back of each picture card)

---

3. Ask: *Which card would you like to discuss [first/next]?*
    a. Ask a participant to pick up card
    b. Restate problem: *This card reminds us there is a problem if…*[state problem]
4. Ask: *Why is this a problem?*
    a. Add information if needed (from key points on back of card)
5. Ask: *Has anyone seen this problem?*
    a. Ask: *What did you see? What did the woman/baby look like?*
    b. Review all signs of problem
6. Start passing card around circle
    a. Ask: *What do you see in the picture that reminds you there is a problem?*
7. Ask: *Is there anything we can do to prevent this problem?*
8. Say: *Let's all find this picture in our booklets*
    a. Check to make sure each woman finds picture
9. Repeat 3-8 for each problem card

---

10. After discussing all cards and placing back in center of circle ask:
    a. *Can we agree that these are problems?*
    b. *Can we agree that if we have any of these problems we will go to a health facility?* Restate problems
    c. *Who should we share this information with?*

Action Cards (1; 3a; 4a)

1. Ask: *What have you seen people in your community do to* _____________ [state topic: *prevent problems during pregnancy/ prepare for birth and complications / prevent problems after birth for baby/mother*]?
    a. Summarize answers
2. Lay down picture cards one by one stating the action it represents (All CAPS statement on back of each picture card)

---

3. Ask: *which card would you like to discuss [first/next]?*
    a. Ask a participant to pick up card
    b. Restate action: *This card reminds us to…* [state action from card]
4. Ask: *Why do we do this?*
5. Ask: *What happens if we don't do this?*
    a. Add information if needed (from back of card)
6. Ask: *What are some things that make it difficult to take this action?*
7. Ask: *What are some things we can do to make these challenges less difficult?*
8. Start passing card around circle
    a. Ask: *What do you see in the picture that reminds you to take action?*
9. Say: *Let's all find this picture in our booklets*
    a. Check to make sure woman finds the picture
10. Repeat 3-10 for each action card

---

11. After discussing all cards ask:
    a. *Can we agree to take these actions to help prevent problems?* Restate actions
    b. *Who should we share this information with?*

GROUP ANTENATAL CARE

Take Action for Healthy Moms and Healthy Babies

Meeting 1:	Meeting 2:	Meeting 3:	Meeting 4:	Meeting 5:
20-24 weeks	24-28 weeks	28-32 weeks	32-36 weeks	36-40 weeks

©Jhpiego Corporation, All Rights Reserved, 2016

1 Preventing problems in pregnancy

REST EVERY DAY, DON'T WORK TOO HARD, SLEEP AND REST UNDER A BED NET AND TAKE IPTP-SP AT EVERY ANTENATAL VISIT

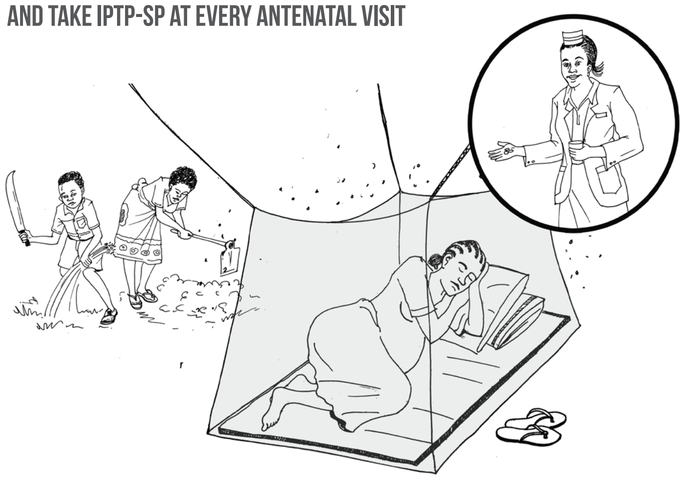

EAT EXTRA MEAL AND DRINK LIQUIDS EVERYDAY. DON'T SMOKE OR DRINK ALCOHOL

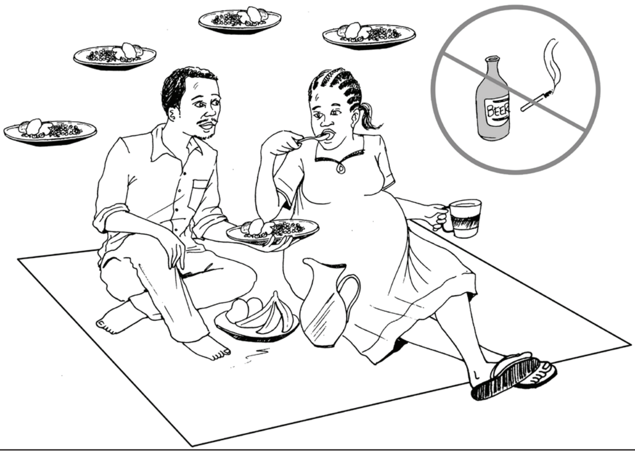

WASH HANDS

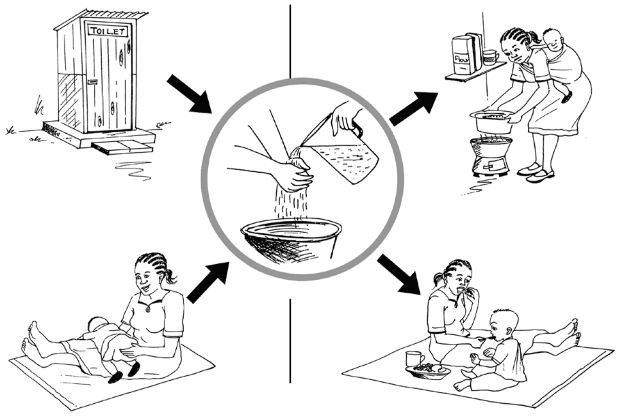

TAKE IRON AND FOLIC ACID

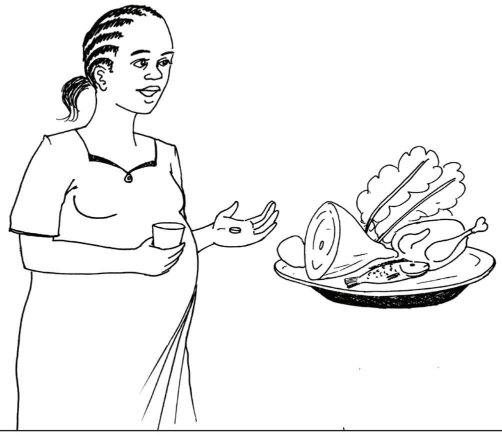

PROTECT AGAINST HIV AND OTHER STIS

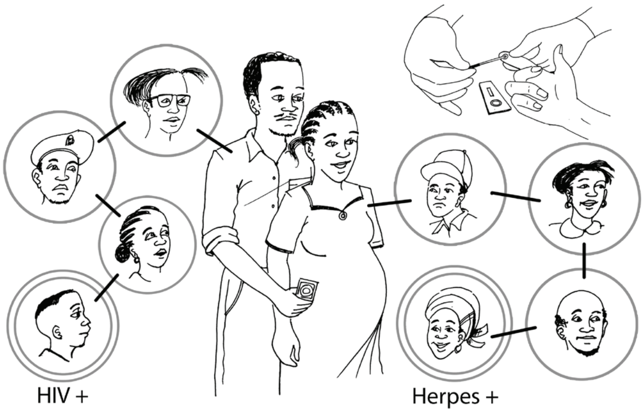

WATCH FOR PROBLEMS, GO TO ANC CLINIC VISITS

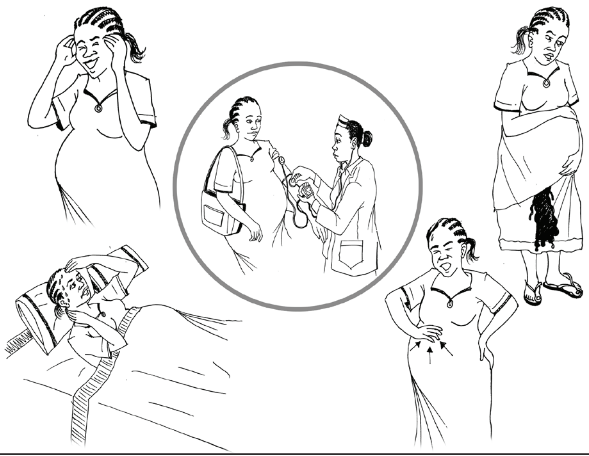

2 Danger signs and problems in pregnancy

PRE-ECLAMPSIA AND ECLAMPSIA: FITS

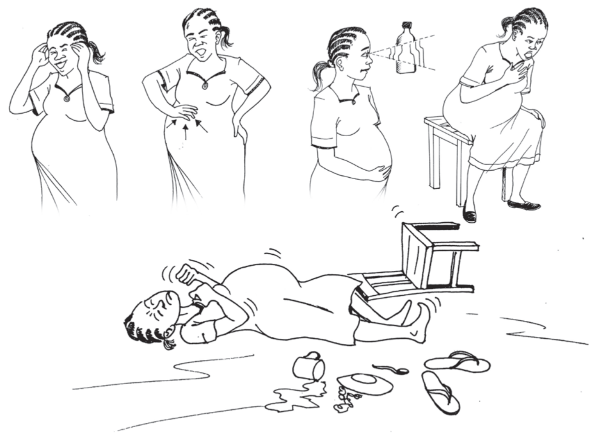

BLEEDING IN PREGNANCY OR TOO MUCH BLEEDING AFTER BIRTH

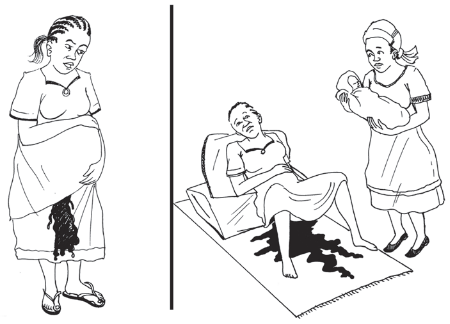

BABY COMING TOO SOON

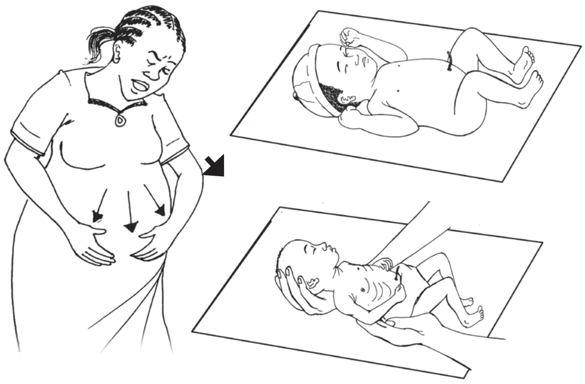

INFECTIONS AND SICKNESS

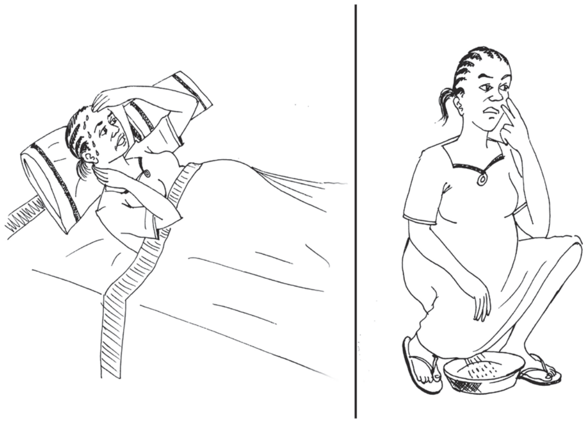

BIRTH DELAY

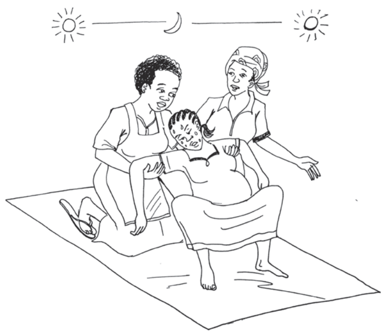

TOO MANY CHILDREN OR MANY PREGNANCIES

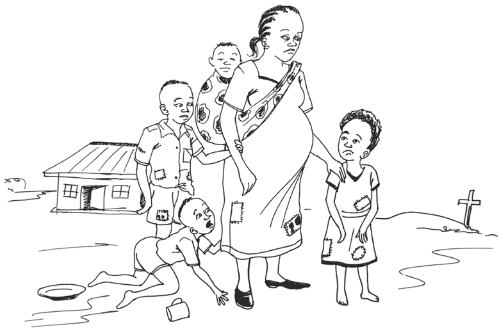

3a Birth Plan

PREPARE A BIRTH KIT

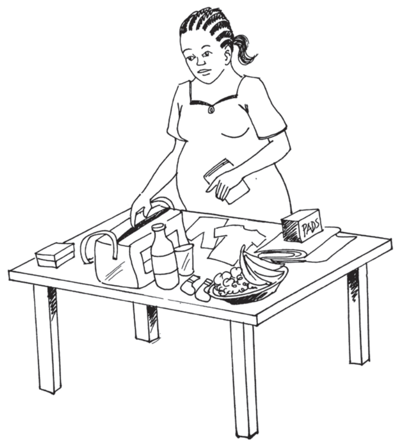

CHOOSE A FACILITY AND SUPPORT PEOPLE

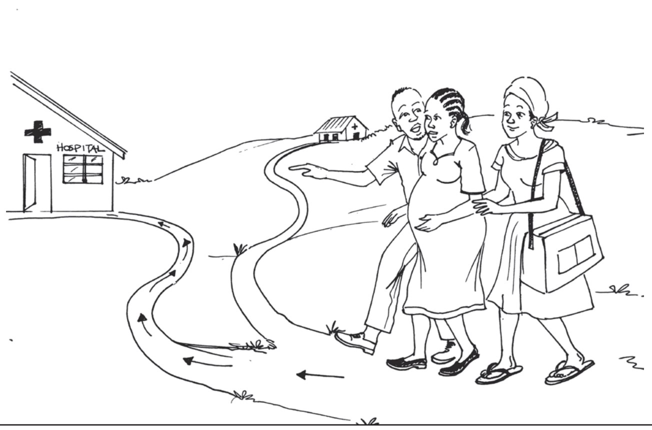

SAVE MONEY FOR BIRTH EXPENSES

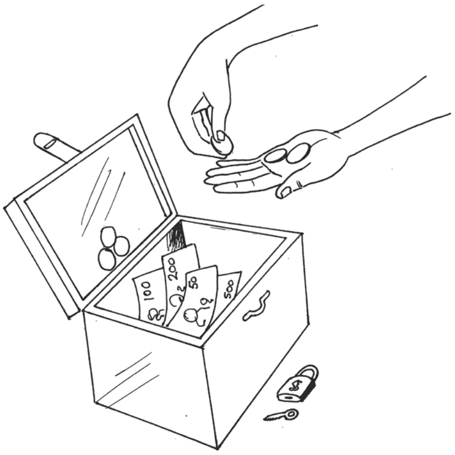

HAVE A TRANSPORTATION PLAN FOR DAY AND NIGHT

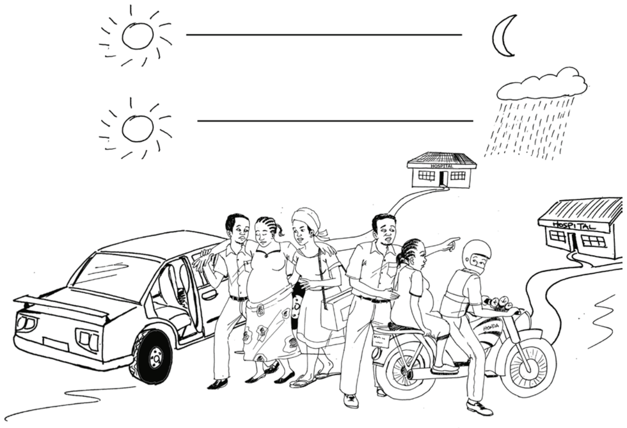

TEACH FAMILY MEMBERS DANGER SIGNS, DISCUSS DECISION MAKING

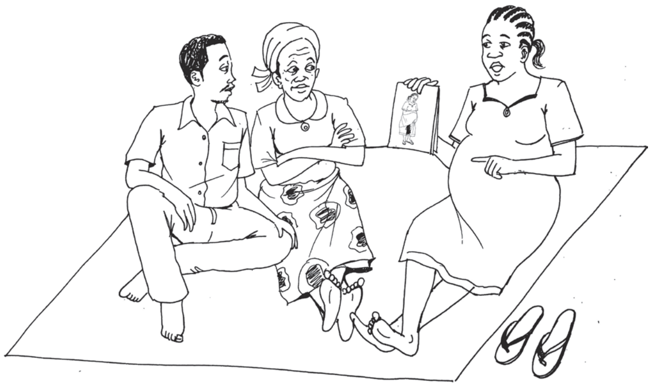

HAVE A PLAN FOR HEALTHY TIMING AND SPACING OF PREGNANCY

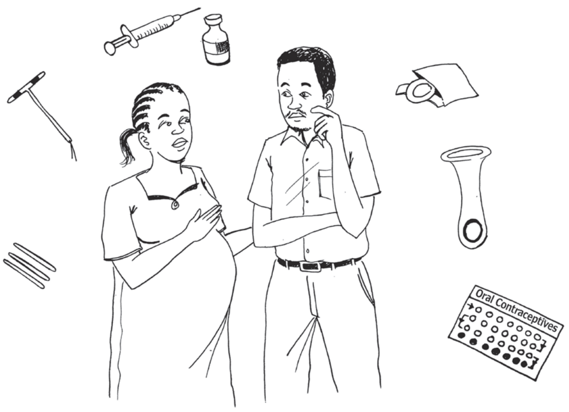

3b Contraception

Options only if you don't want more children

TUBAL LIGATION

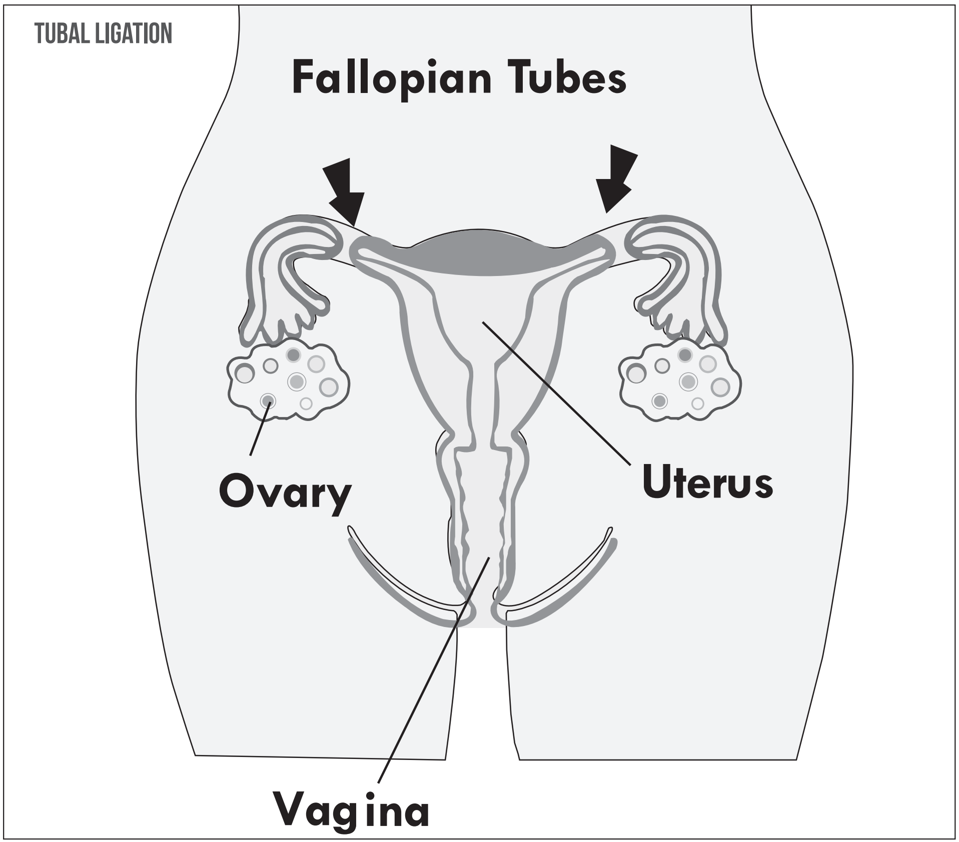

VASECTOMY

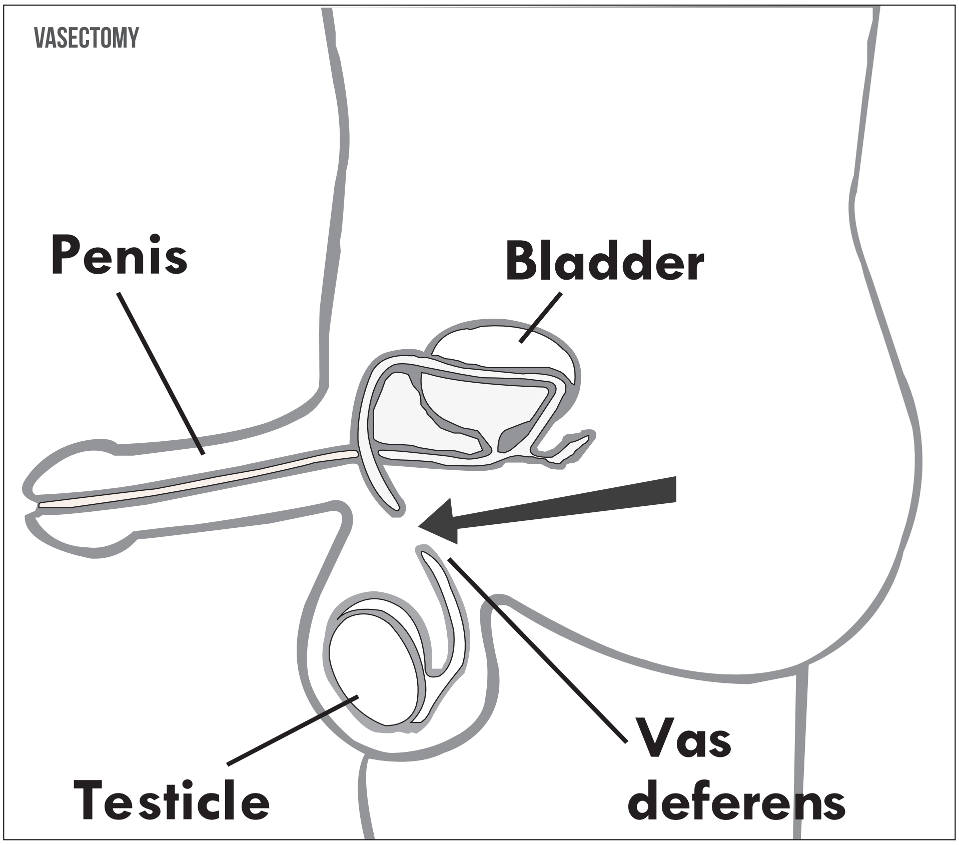

3b Contraception

Options if you are not breastfeeding

PROGESTIN-ONLY INJECTABLES

CAN START IMMEDIATELY IF NOT BREASTFEEDING; START AT 6 WEEKS IF BREASTFEEDING

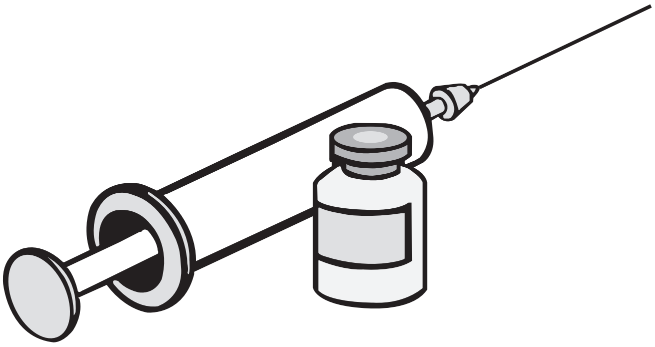

MONTHLY INJECTABLE

CAN START 3 WEEKS AFTER BABY BORN IF NOT BREASTFEEDING; 6 MONTHS AFTER BABY BORN IF BREASTFEEDING

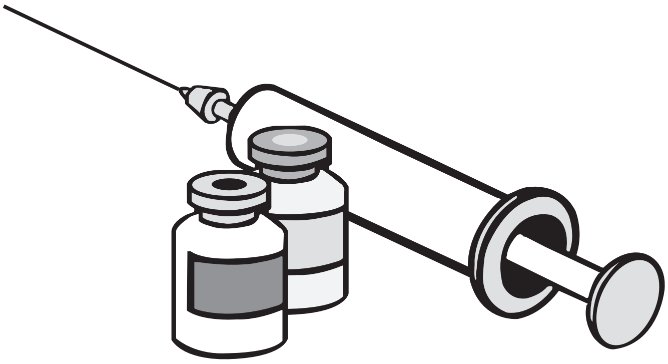

THE PILL

CAN START 3 WEEKS AFTER BABY BORN IF NOT BREASTFEEDING; 6 MONTHS AFTER BABY BORN IF BREASTFEEDING

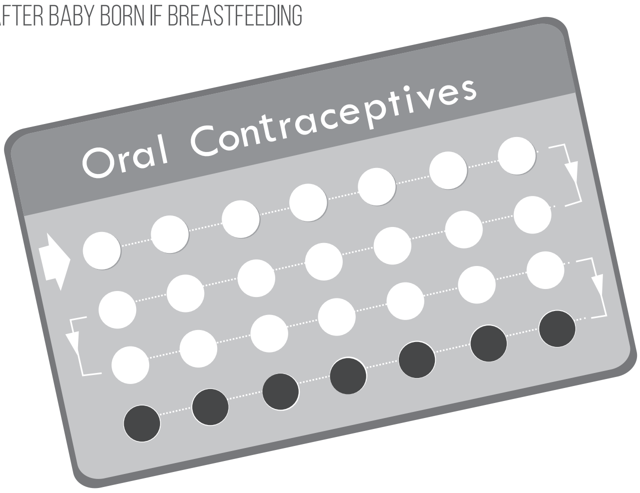

3b Contraception

Group C: Breastfeeding

HORMONAL OR COPPER IUD

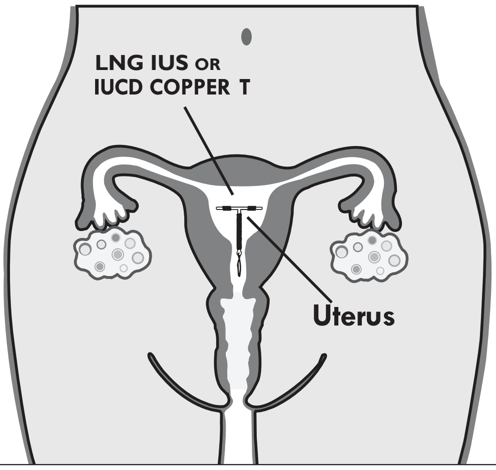

HORMONAL IMPLANTS

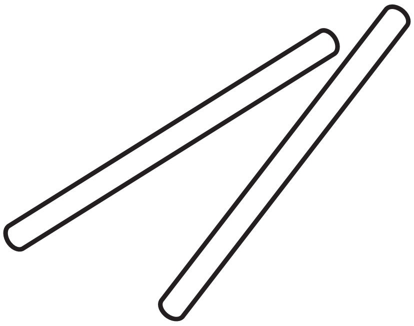

LACTATIONAL AMENORRHEA METHOD

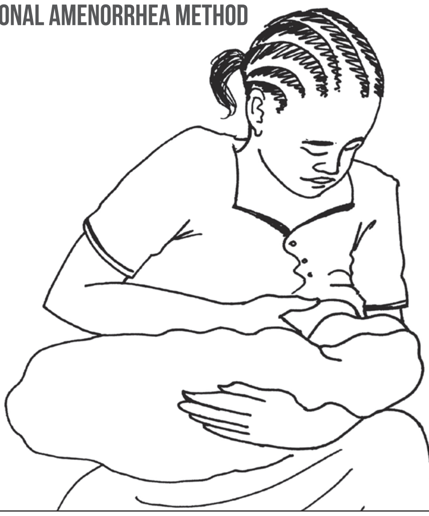

MINI PILL

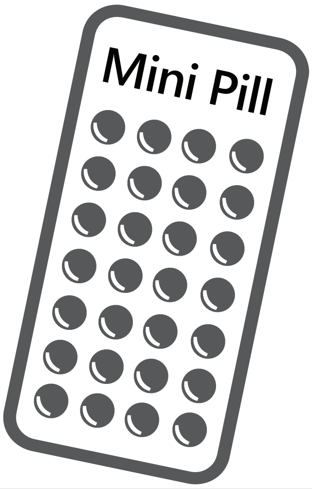

CONDOM

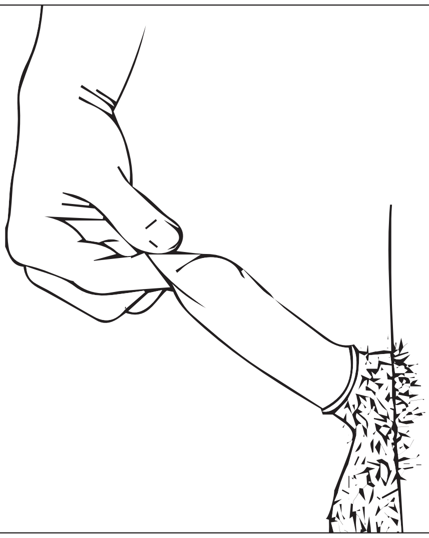

WITHDRAWAL

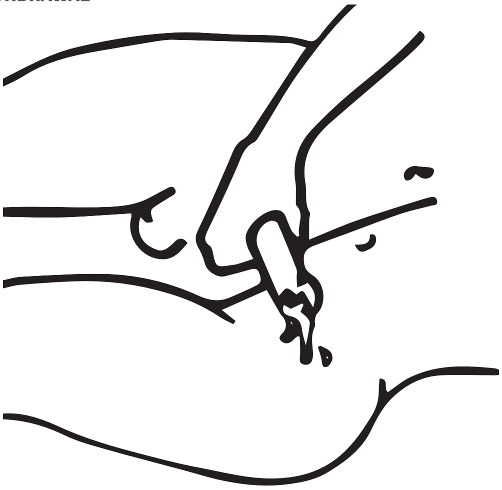

4b Lactational amenorrhea method (LAM)

GIVE BABY ONLY BREASTMILK, DAY AND NIGHT

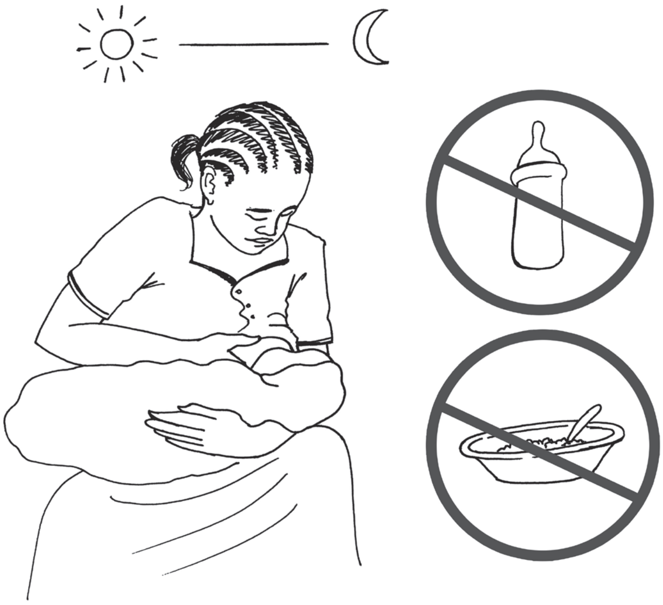

NO MENSTRUAL BLEEDING

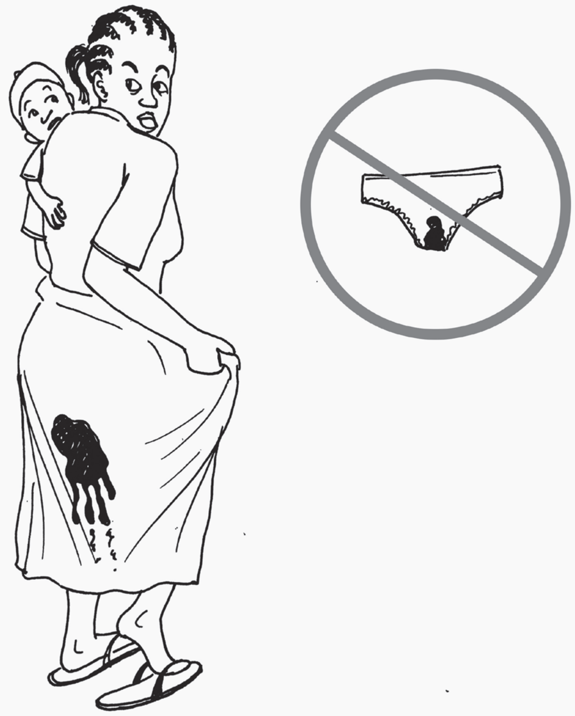

BABY IS LESS THAN 6 MONTHS OLD

0 - 6 months

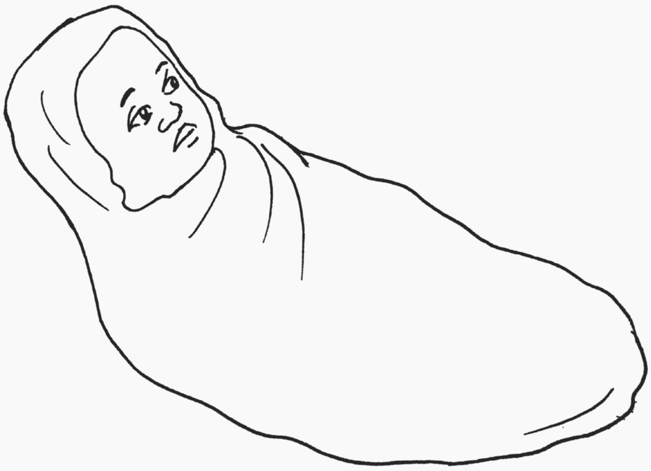

PLAN FOR NEW METHOD BEFORE BABY IS SIX MONTHS AND BEFORE BABY EATS OR DRINKS ANYTHING OTHER THAN BREASTMILK

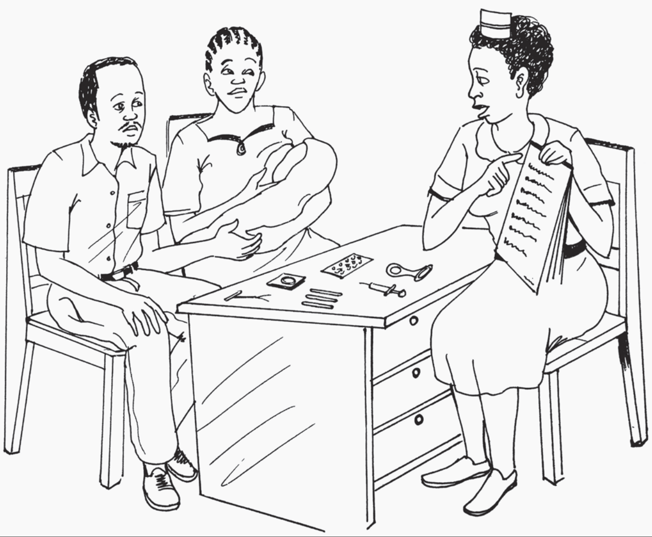

4a Preventing problems after birth

BREASTFEED IMMEDIATELY AND OFTEN. GIVE ONLY BREASTMILK FOR 6 MONTHS

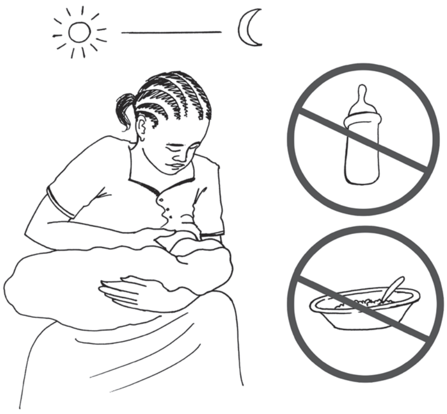

KEEP BABY WARM

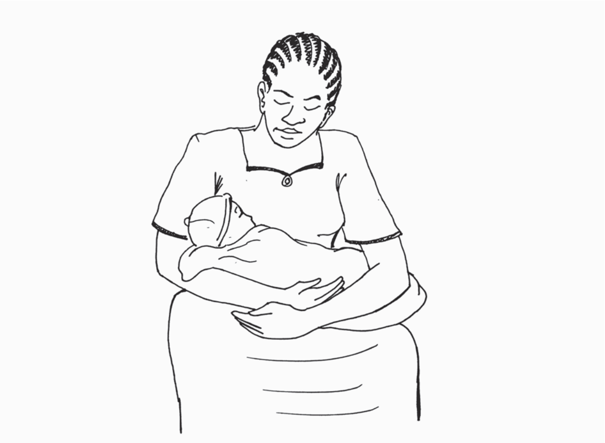

KEEP MOTHER, BABY AND CORD STUMP CLEAN

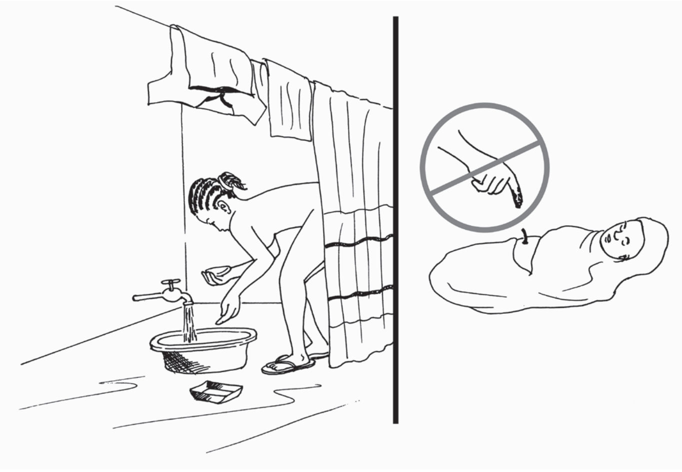

DRINK LIQUIDS, EAT AT LEAST 4 TIMES A DAY

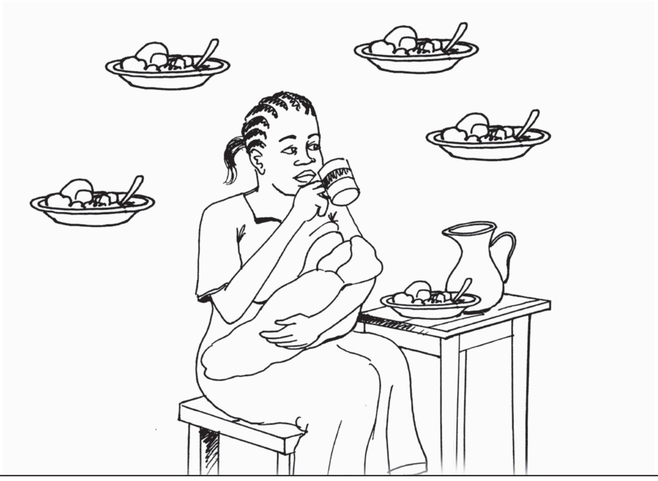

NO WORK OR LIFTING FOR 2 WEEKS, BABY SLEEPS WITH MOTHER UNDER BED NET

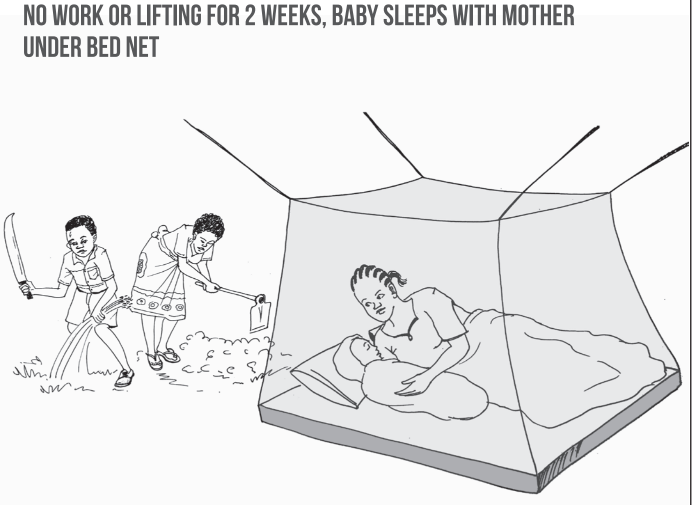

WATCH FOR PROBLEMS, GO TO PNC CLINIC VISITS DAY 1, 3, 7 AND 6 WEEKS

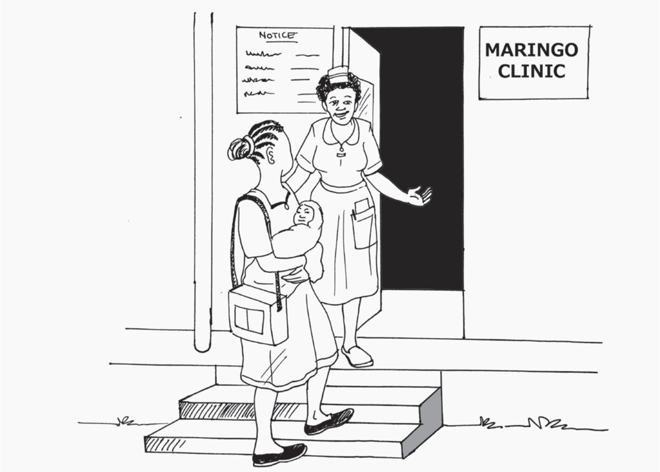

5a Baby problems

FAST BREATHING OR TROUBLE BREATHING

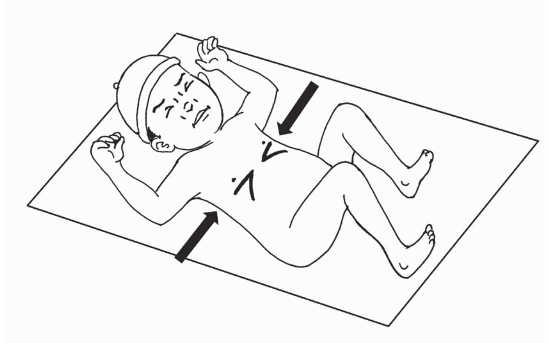

TOO WARM OR TOO COLD

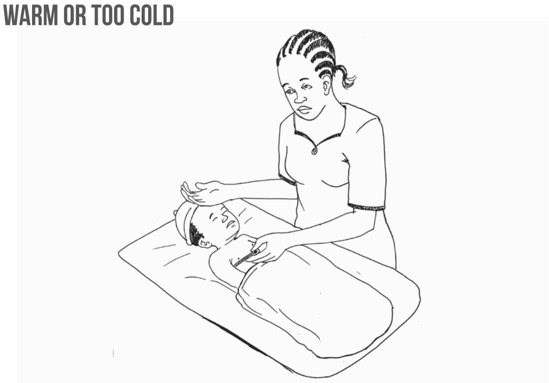

MOVES DIFFERENTLY: FITS/CONVULSIONS OR BECOMES LESS ACTIVE

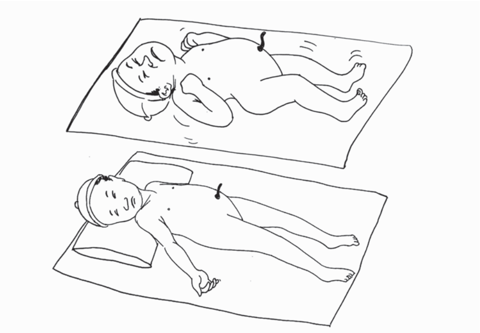

BABY STOPS FEEDING WELL

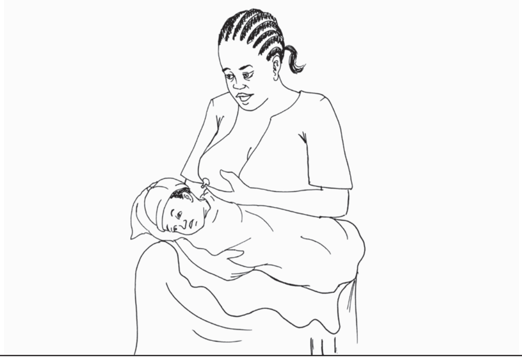

BABY TOO SMALL

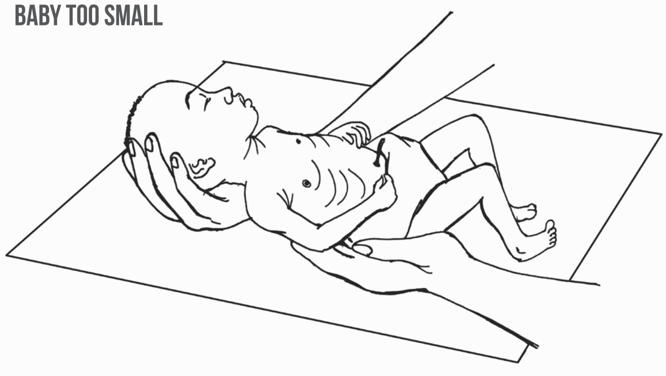

UMBILICAL CORD IS RED, HOT, OR BLEEDING

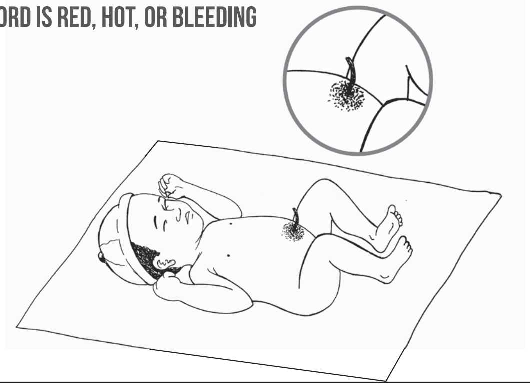

BABY IS YELLOW

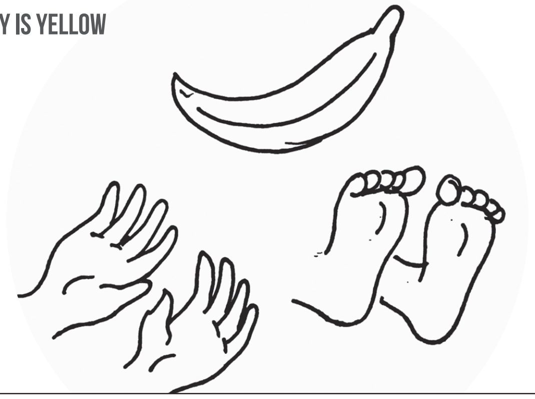

5b Woman problems after birth

TOO MUCH BLEEDING

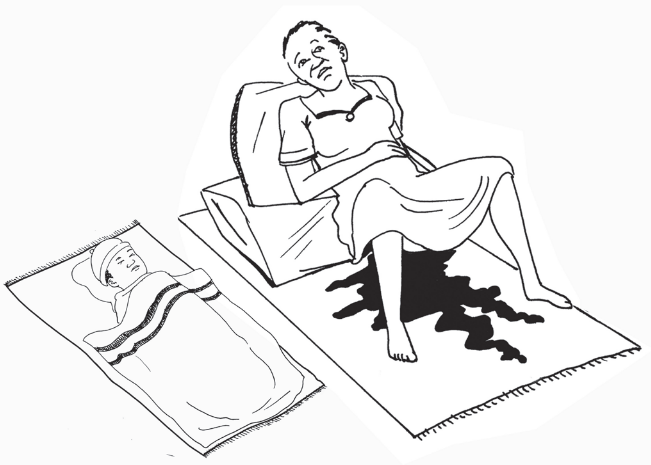

ANY SEVERE PAIN: ABDOMEN, BREASTS, CHEST, LEGS, OR HEAD

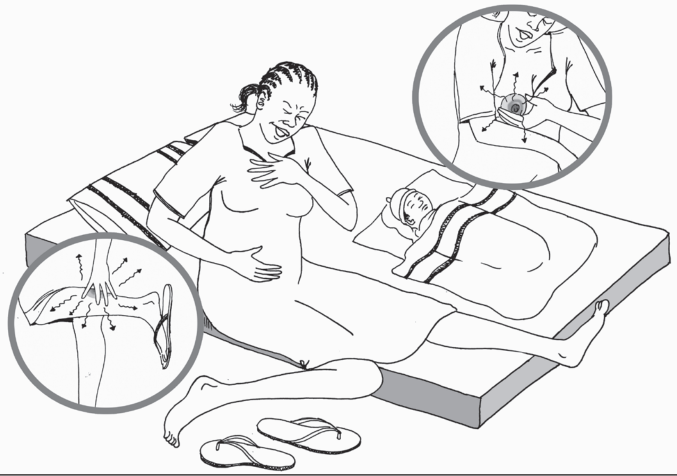

FITS: ECLAMPSIA

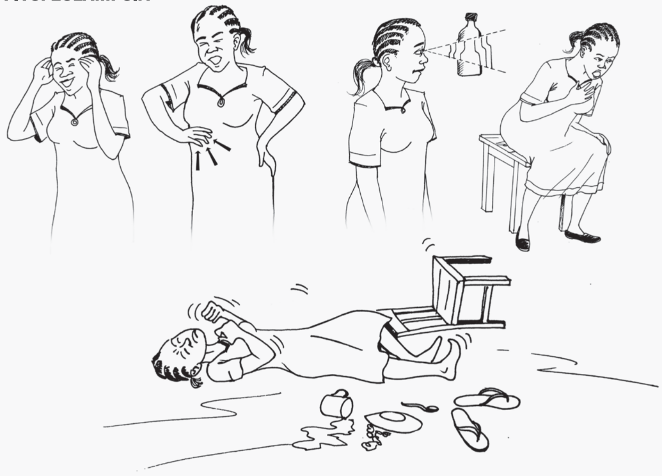

FEVER OR CHILLS, FEELING VERY ILL

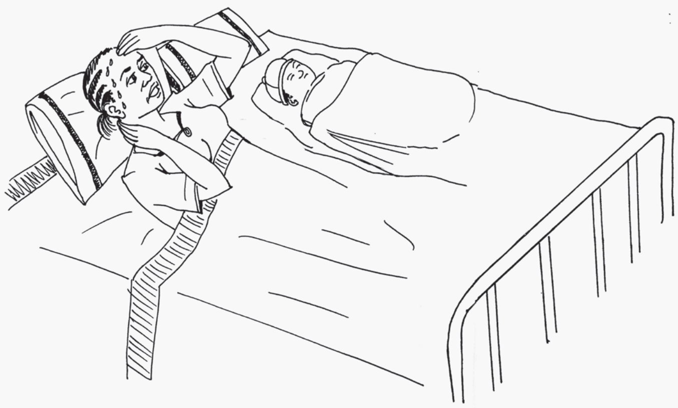

DIFFICULT OR PAINFUL URINATION

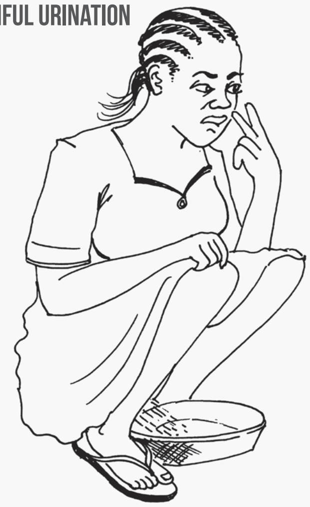

RED OR HOT BREASTS

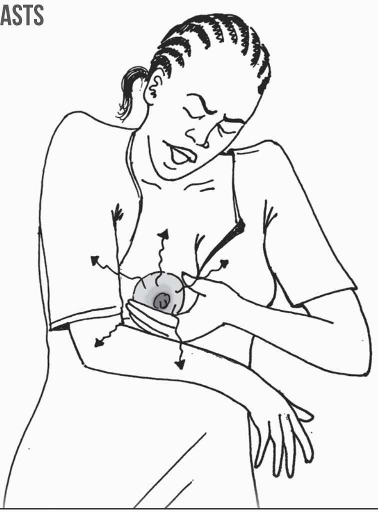

Ready for baby!

- ☐ Birth Facility chosen
- ☐ Transportation ready
- ☐ Support people picked
- ☐ Money saved
- ☐ Birth kit ready
- ☐ Plan for healthy timing and spacing of pregnancy
- ☐ Plan for care after baby born (PNC)
- ☐ Warm clothes/blankets for baby ready
- ☐ Know danger signs and how to prevent problems
- ☐ Baby name chosen
- ☐ First outfit ready
- ☐ Family ready for baby
