# Supplementary material for: Transforming women’s and providers’ experience of care for improved outcomes: A theory of change for group antenatal care in Kenya and Nigeria
Source: PLoS One. 2022 May 3;17(5):e0265174. doi: 10.1371/journal.pone.0265174 (PMC9064109; doi:10.1371/journal.pone.0265174)
Supplement: S2 Appendix — (PDF) [file pone.0265174.s002.pdf]

Quality Assurance tools

The following quality assurance tools were created for the study to be used by study program officers for mentoring and coaching purposes, and by G-ANC facilitators (providers) for self-reflection and debriefs with each other. Program officers attempted to attend 80% of meetings for the first two cohorts, with reduced frequency thereafter dependent on facilitator skill and comfort. Facilitators were trained and strongly encouraged to hold brief debriefs with each other utilizing these tools after every meeting.

1. Co-Facilitator Debrief
    a. Designed to mimic a coaching methodology, reflecting on strengths, learning, and future goals for specific sections of each meeting
2. Fidelity Checklist
    a. Intended as a quick checklist to evaluate fidelity to key components of the model
    b. Checklists were collected and examined as programmatic data for quality assurance and improvement, not as part of study protocol
3. Individual Reflection
    a. Self-reflective tool focused primarily on facilitation skills, also used by mentors as a guide for feedback

Co-Facilitator debrief

Complete this with your co-facilitator immediately after each meeting. Keep debriefs together with the group ANC materials. Refer to this before you lead the next meeting to remind yourselves what you wanted to try or do differently in the next meeting.

Meeting #: ______________________________

Group cohort (name/#):__________________

Number who attended this meeting: _______

Number of women in group: _____________

Facilitators:

Total Time of meeting:___________________

Women reminded via phone or text prior to meeting? Yes_______ No_______

Program Officer present? Yes______ No___

Guests present? Yes_______ No_______

Planning before the group meeting

What went well?

What did you learn?

What will you try differently next time?

Assess and check in

What went well?

What did you learn?

What will you try differently next time?

Review, Learn and Practice

What went well?

What did you learn?

What will you try differently next time?

Reflect and plan

What went well?

What did you learn?

What will you try differently next time?

Overall

Suggestions for changes to this meeting:

Were any activities skipped? Which activities and why?

Fidelity checklist

**Fidelity scale items**<br>*For items with 1-5, rate, where 1=strongly disagree and 5= strongly agree*	**Yes**	**No**	**Comments**
No more than 15 women in group			
Same facilitors(s) as previous meeting with this group			
Group conducted in a circle			
Facilitator sat with group and did not stand while conducting meeting			
Facilitator used key facilitation skills: empowering questions, encouraged participation by all, paraphrasing, validation, summarizing, and active listening	1 2 3 4 5		
Meeting was highly participatory, the women spoke more than the facilitator	1 2 3 4 5		
Time was given to discuss questions/concerns/topics identified by the women (e.g. from basket)			
All activities in guide were done	1 2 3 4 5		
Private consultations were provided in the group space			
Women completed self-assessments			
Ritual activities done to start Circle up; and close meeting			
Sister check ins were done at start of meeting and sister planning at end of meeting			
Picture cards passed around so women could handle			
All picture cards and Key messages from illustrations were covered	1 2 3 4 5		
Time provided for socialization			
All Group materials were available			

Individual reflection

This is for your own use, to help you reflect and improve on your facilitation of group ANC. You may wish to get feedback from your co-facilitator or the program officer, or you may choose to do this as a private activity

**Today…**	**Rate on a scale of 1-5**<br>**1=strongly disagree**<br>**5=strongly agree**
I was able to get the women to actively participate in group discussions and activities	1 2 3 4 5
I tried to engage quieter members, and respectfully managed anyone who was dominating conversation	1 2 3 4 5
I picked up on how the women in my group were feeling or thinking and responded	1 2 3 4 5
I effectively managed time and kept focus on key topics while still responding to women's interests and needs	1 2 3 4 5
I encouraged women to answer each others questions rather than always providing the answer myself	1 2 3 4 5
If controversial practices were brought up I facilitated conversation among all women about their experience and knowledge and let group members identify potential problems	1 2 3 4 5
I did not read the facilitators guide out loud during the meeting	1 2 3 4 5
I had good time management	1 2 3 4 5
I used the illustration cards to facilitate discussion around what women knew; barriers and solutions to actions; and to reach agreement	1 2 3 4 5
Key content on the illustration cards was covered (but not read)	1 2 3 4 5
I gave clear directions for activities, and offered support and feedback during any practice	1 2 3 4 5
