# Supplementary material for: Transforming women’s and providers’ experience of care for improved outcomes: A theory of change for group antenatal care in Kenya and Nigeria
Source: PLoS One. 2022 May 3;17(5):e0265174. doi: 10.1371/journal.pone.0265174 (PMC9064109; doi:10.1371/journal.pone.0265174)
Supplement: S3 Appendix — (PDF) [file pone.0265174.s003.pdf]

Qualitative tools

The following guides were created for the study for the purposes of guiding qualitative data collection. Data collection was conducted in one of five languages: English, Kiswahili, Dholuo (Luo), Kamba, or Hausa by native speakers, all of whom were also fluent in English. Focus group facilitators and interviewers were oriented to the research questions and trained by author PW preceding data collection. Guides were not translated in print.

1. ANC focus group guide: Service Providers
   a. This guide was used for both FGD and interviews
2. ANC focus group guide: Women and Addendum
   a. Both guides were used with recently delivered women
3. ANC in-depth interview guide: Women
   a. See section headings for questions asked to specific sub-groups

ANC focus group guide: Service providers

Study title: Cluster randomized control trial to evaluate Group Antenatal Care (G-ANC) compared with standard individual care in Kenya and Nigeria
PI: Jeff Smith

Health Facility code: ___________
County/LGA: ________________

Date of focus group:___________

First and Last Name of data collector:_______________________________

1. What were your experiences in conducting Group ANC sessions with the women in your PHC?
    a. What did you enjoy most about providing care using Group ANC?
    Probe
        i. While facilitating
        ii. In providing post-group care?
    b. What did you least like about providing care using Group ANC?
        i. While facilitating
        ii. In providing post-group care
    c. What was most surprising about providing care in this manner?
2. How would you describe the women in your group (personality differences and its impact on group dynamics)? How would you describe your relationship with them? Did women in the group get along with each other? Did you get along with them? How well did they work together as a group?
    a. How does this differ from the way you feel about or interact with women when offering standard individual care?
    b. Has it changed the way you think about your patients?
    Probe
    i. Are you more concerned about the level of care they get
    ii. About the way you address and respect each person's individuality?

3. How did participating in group care change the way you view or feel about your job?
    a. Are there ways the experience of G-ANC changed your expectations for the future? Tell me more about that.

4. What would you say to an administrator (in-charge) who wanted to implement group based care [in various facilities and in your specific facility]?
    a. What are the reasons to do it?
    b. What are the reasons not to do it?
    c. Describe the context where it would work best – what does that facility and clientele look like? What needs to be in place for it to be successful?

5. What would you say to a Policy maker (SPHCDA Official) who wanted to implement group based care [in various facilities and in your specific facility]?
    a. What are the reasons to do it?
    b. What are the reasons not to do it?
    c. Describe the context where it would work best – what does that facility and clientele look like? What needs to be in place for it to be successful

6. Are there any changes you would suggest or topics you would like to see added to the G-ANC sessions?
    a. To make it easier or more enjoyable for providers?
    b. To make it easier or more enjoyable for patients?
    c. Timing changes?
    d. Training for Service providers?

7. What would you like to tell other facilities about providing group antenatal care?

ANC focus group guide: women

Study title: Cluster randomized control trial to evaluate Group Antenatal Care (G-ANC) compared with standard individual care in Kenya and Nigeria
PI: Jeff Smith

Health Facility code: ____________
County/LGA: ________________

Date of focus group:___________

First and Last Name of data collector:_________________________________

1. What would you like to tell others about receiving antenatal care in a group like this?
    a. What did you like best about receiving care in this manner?
    b. What did you like least about receiving care in this manner?

2. For those of you who have attended individual care before, how would you compare the two forms of care or describe them to a friend trying to choose between the two?
    a. What has your group, or this manner of care meant to you?

3. Describe how you feel about the group facilitator and ANC provider and your relationship with them
    a. Probe: feel respected? Trust them? Feel heard/seen as a person?
    b. How does this differ from experiences you've had with the health system or other providers in the past?

4. Describe how you feel about other women in your group and your relationship with them

5. Thinking back to the beginning of your pregnancy, before you joined the group, has participation in the group changed your mind or intention about anything?
    a. Probe: For example, where you plan to deliver, use of post partum family planning, breastfeeding/infant feeding practices
        1. Tell me more about that
    b. How has group participation changed behaviors related to your health?

6. Thinking back to the beginning of your pregnancy, before you joined the group, has participation in the group changed the way you think about your pregnancy or your baby?

    a. What about how you see yourself or how you want to be in the world? (self-esteem)
    b. Do you think you are more likely to ask questions or speak up about things that impact you or your baby's health?
    c. What about making decisions that impact your health?
        1. How will you make those?
        2. Is that different that how you would have made them at the beginning of your pregnancy?
        3. Has participation in your group changed how you think about making decisions and taking action? How?
7. Are there any changes you would suggest or topics you would like to see added?

ANC focus group guide: women

Study title: Cluster randomized control trial to evaluate Group Antenatal Care (G-ANC) compared with standard individual care in Kenya and Nigeria
PI: Jeff Smith

Health Facility code: ___________
County/LGA: _______________

Date of focus group:__________

First and Last Name of data collector:______________________________

1. A pregnant woman of 20-24 weeks:
   a. What is her name? How old is she? Has she had children before or is she a first time mother? Did she go to school? Does she live or have constant contact with her mother-in-law?
   b. ***Amina*** wants to enroll for ANC:
      i. Would you advise Amina to go to your health facility? Why?
      ii. Do you think your good/not so good experiences with the G-ANC would influence Amina's desire to attend G-ANC?
      iii. Should Amina attend the group sessions or the individual sessions at the clinic? Why? In what way has G-ANC benefited you?
      iv. What is her experience like with the facilitator and health providers?
         1. Kind, gentle, answer questions she had, allow her to make contributions or draw her into conversations? Experience with health staff during delivery- beat, shout at etc)?
         2. Was her experience at the G-ANC different from what she had experienced before?
      v. What was Amina's relationship with her group? Did she feel accepted, involved in the group? Did they get in touch with her after meetings; remind her of upcoming meetings, could she contact them if she had issues? What was the means of communication mostly used?
      vi. How has what she learnt in G-ANC changed Amina's behaviours related to her health and what she thinks about what to get done around her pregnancy and delivery period?
         1. For example, what she eats, understanding of danger signs, where she plans to deliver, use of post-partum family planning, breastfeeding/infant feeding practices
      vii. Has G-ANC changed the way Amina was at the beginning of her pregnancy?
         1. Is she able to better express her health needs or her baby's health needs to her partner/mother-in-law?
         2. Is she more likely to ask questions or speak up about her or her baby's health
         3. Does she receive better support with her group than with individual care? (Can she communicate with other women about

specific issues she might be facing at home or with her pregnancy/ Family planning decisions and seek advice? Does she feel safe doing this?)

4. Is she better able to make/negotiate decisions or take actions towards her safe delivery? How?
    a. Are they different from what she would have made at the beginning of her pregnancy?
    b. How will she go about making these decisions?

2. Are there any changes you would suggest or topics you would like to see added to Amina's G-ANC sessions?

ANC in-depth interview guide: women

Study title: Cluster randomized control trial to evaluate Group Antenatal Care (G-ANC) compared with standard individual care in Kenya and Nigeria
PI: Jeff Smith

Health Facility code: ___________
County/LGA: _______________

Date of in-depth interview: __/___/_____ (dd/mm/yyy)

First and Last Name of data collector:______________________________

(All in-depth interviews (IDIs) will carried out as per the IDI procedure that will be described in the study manual and will be audio recorded after obtaining verbal permission from the participants. Participants will not use their real names.)

Questions for all participants

1. Can you tell me about your experience in group ANC?
    a. What did you like best about receiving care in this manner?
    b. What did you like least about receiving care in this manner?
    c. What was most surprising about receiving care in this manner?
    d. Would you advise your friends to receive care at your health facility? Why?
    e. Would you advise your friends to attend the group antenatal sessions or the individual ANC sessions? Why? What will you tell them?
    f. Do you think your good/not so good experiences with the G-ANC will influence your friend's decision to attend the G-ANC? How and why?
    g. Would you encourage your friends to come for all the group meetings or only for some meetings? Why? (Probe for lessons learned at each meeting session)
2. Did you attend antenatal care in previous pregnancies? How many previous deliveries have you had and where did you deliver the last pregnancy?
    a. What influenced your decision to have your babies at home in previous pregnancies?
    b. What influenced your decision to have your baby in the hospital in this pregnancy?
3. Did you come for all the group ANC meetings? Compare between the two forms of care and how you would describe them to a friend trying to choose between the two.
    a. What did you benefit from the group ANC sessions?
4. What was your experience like with the facilitator and health providers?
    a. Kind, gentle, answer questions you have/had? Allows you to make contributions or draws you into group conversations? Experience with health staff during delivery- beat, shout at etc)?

   b. Was your experience at the G-ANC different from what you had experienced before?
5. What was your relationship with your group like? Did you feel accepted by your group and involved in the group? Did your group members get in touch with you after group meetings to check on you, remind you of upcoming meetings? Could you contact them if you have issues? What was the means of communication mostly used? Did they contact you when they had issues and ask for your advice?
6. Thinking back to the beginning of your pregnancy, before you joined the group, has participation in the group changed your mind or intention about anything?
   a. Probe: For example, what you eat, understanding of danger signs, where you planned to deliver, use of post-partum family planning, breastfeeding/infant feeding practices. Tell me more about that
   b. How has group participation changed behaviors related to your health?

7. Thinking back to the beginning of your pregnancy, before you joined the group, has participation in the group changed the way you think about your pregnancy or your baby?
   a. Has G-ANC changed the way you were at the beginning of your pregnancy?
   b. Is she able to better express her health needs or her baby's health needs to her partner/mother-in-law?
   c. Is she more likely to ask questions or speak up about her or her baby's health at the health facility
   d. Does she receive better support with her group than with individual care? (Can she communicate with other women about specific issues she might be facing at home or with her pregnancy/ Family planning decisions and seek advice? Does she feel safe doing this?)
   e. Is she better able to make/negotiate decisions or take actions towards her safe delivery? How?
   f. Are they different from what she would have made at the beginning of her pregnancy?
   g. How will she go about making these decisions?
   h. What about making decisions that impact your health?
      - How will you make those?
      - Is that different that how you would have made them at the beginning of your pregnancy?
      - Has participation in your group changed how you think about making decisions and taking action? How?
8. Are there any changes you would suggest or topics you would like to see added?

ANC in-depth interview guide: women

Study title: Cluster randomized control trial to evaluate Group Antenatal Care (G-ANC) compared with standard individual care in Kenya and Nigeria
PI: Jeff Smith

Additional questions for women who did not continue with group ANC

(*These questions are intended for women who 1) came to only one or two group meetings during their pregnancy, AND 2) we do not have a reason from the monitoring data for why they stopped attending the group meetings. If there is an obvious reason such as preterm delivery or a known medical complication, there is no reason to ask these questions.*)

9. Some women attend group meetings throughout their entire pregnancy, while other women only attend one or two meetings. How many meetings were you able to attend? [If the woman attended meetings throughout her pregnancy, do not ask any further questions in this section]
10. Would you have liked to attend the meetings throughout your pregnancy? Were you benefiting from the meetings? Why or why not?
11. What things made it difficult for you to continue attending the meetings?
12. Could anything have been done to make it easier for you to attend the meetings?
    a. What are some reasons why women continue to attend group ANC until the end?
    b. What are some reasons why some women stop attending?
    c. Is it something that you do in your area? Is this is how it's done around here? Haka kuka saba yi?

Additional questions for women who attended group ANC, but did not deliver in a health facility

(*These questions are intended for women who attended Group ANC, but then did not deliver in a health facility.*)

13. After attending the group meetings, many women make a plan to deliver their babies in a health facility. However, others choose to deliver their baby at home. Did you deliver your baby in a health facility, or at home? [If the woman delivered her baby in a health facility, do not ask any further questions in this section]
14. Had you planned to deliver your baby in a health facility? If yes, what things prevented you from delivering your baby in a health facility?
15. Had you planned to delivery your baby at home? If yes, what things made you to decide to deliver your baby at home?

Additional questions for women who experienced complications during pregnancy or labor and delivery

(*These questions are intended for women who experienced a complication during pregnancy, labor or delivery such as premature labor or a danger sign like heavy bleeding. We want to know if participation in Group ANC provided them with resources that helped them to respond to the problem*)

16. Did you have any health problem or problem with your pregnancy that required you to seek care during your latest pregnancy? [If the woman reports no problem, do not ask any further questions in this section]
17. Can you tell me about the problem you experienced during pregnancy or during labor and delivery? How did you notice you had a problem? What kind of a problem was it?
18. Who did you talk to when you were deciding what to do about the problem? What options or sources of care did you consider?
19. Where did you end up seeking care? Did the discussions in the group meetings give you any ideas about where to seek care?
20. What type of transport did you take when you sought care? Did the discussions in the group meetings give you any ideas about what kind of transport to take, and how to access the transport?
21. How did you pay for the care? Did the discussions in the group meetings give you any ideas about how to pay for the care?
