# Supplementary material for: Transforming women’s and providers’ experience of care for improved outcomes: A theory of change for group antenatal care in Kenya and Nigeria
Source: PLoS One. 2022 May 3;17(5):e0265174. doi: 10.1371/journal.pone.0265174 (PMC9064109; doi:10.1371/journal.pone.0265174)
Supplement: S1 Table — (DOCX) [file pone.0265174.s004.docx]

**S1 Table: Baseline demographic characteristics of enrolled ANC providers at intervention sites.**

|  | **Nigeria (n=39)** | **Kenya (n=36)** |
| --- | --- | --- |
| **Mean Age (range)** | 35 (21, 64) | 36 (24, 54) |
| **Sex** |  |  |
| Male | 2 | 6 |
| Female | 37 | 30 |
| **Mean Years Providing ANC (range)** | 8.1 (1, 32) | 9.0 (1, 30) |
| **Designation*** |  |  |
| Midwife | 10 | 1 |
| Nurse | 7 | 34 |
| CHEW | 24 | 1 |

In Nigeria enrolment numbers include nine providers who did not facilitate G-ANC and were not included in the endline survey. Most of these were supervisors. Results in Kenya include six providers who were trained and enrolled after death of one study participant transfer of five others.

* multiple selections allowed
