# Supplementary material for: Transforming women’s and providers’ experience of care for improved outcomes: A theory of change for group antenatal care in Kenya and Nigeria
Source: PLoS One. 2022 May 3;17(5):e0265174. doi: 10.1371/journal.pone.0265174 (PMC9064109; doi:10.1371/journal.pone.0265174)
Supplement: S2 Table — (DOCX) [file pone.0265174.s005.docx]

**S2 Table. Illustrative quotes discussing G-ANC impact on health knowledge and health promoting behavior within and beyond women’s households.**

|  | **Within household** | **Beyond household** |
| --- | --- | --- |
| **Recognition and response to danger signs and symptoms** | *I started experiencing the drainage almost one month and some days before delivery. Immediately I called the matron and she told me to rush to the hospital. She called one of our nurses who helped me in the hospital. But before then, in the booklet they gave us, drainage and other danger signs and what to do was there and we were taught. I explained to my husband, and since I explained, he started to help me (Woman, NG)*  *Last year, I got a baby, but I had not been trained well…I lost that baby because he was suffering from pneumonia. I did not know how to check the temperatures; I did not know how to check his breathing. Now I know what to do, to keep the baby well (Woman, KE)* | *Right now, I know more than I knew before…when a child is sick, I have to take him or her to the hospital even though my husband is not around. Even there was my neighbors’ child who was sick, and the mother did not know what was wrong. He was convulsing, he had high fever. She had already left the child there, that he was dying, and she was crying. I told her that no, the child had not died, let me call boda boda [motor bike] to come and pick you and take you to [the health facility]. The baby has high fever, remove those many clothes and rush to the hospital…The child is alive to date…Before, I did not know that I could not advise such a woman to help a child. Back then, I would have helped her cry that the child had already died (Woman, KE)* |
| **Understanding and enacting health promoting behaviors** | *Personally, in my first pregnancy I used to throw the tablets [IFAS] because I did not know why they gave us [other respondents laughing], I did not know they were important, but here we were trained on their importance and I remained loyal to that medication up to the last minute (Woman, KE)*  *G-ANC has helped us. Our other children, we gave them water, but when we came, we were advised not to give our babies water, breastfeed them exclusively, feed them well…We did that, and we see its usefulness. The hotness of the body has reduced and no more diarrhea and rashes…[my daughter] is more active than my other children when they were her age (Woman, NG)* | *I helped my friend, first time mum, she did not know how to position baby when breastfeeding. She called me when the baby was crying….we had been taught that when you breastfeed the baby its stomach and yours should touch each other. So, I told her to hold the baby that way and breastfeed and the baby got helped. (Woman, NG)* |
| **Advocating for behavior change and care-seeking** | *We save money right from when the pregnancy is early… get your “asusu” (safe box) and gather your money little by little. … if he says “I don’t have money, so you are not going to the hospital to deliver” then you say “I have money” my money is in that place, take it and take me to the hospital. (Woman, NG)*  *You know some parents or husbands - you see the training materials that we were using, we could show them and make them understand. … before it was hard to tell them anything. For example, if it is your mother, what do you tell her - she is the one who gave birth to you. But with these materials she is able to tell that you have learned and gained new knowledge, so she is able to believe what you tell her. (Women, KE)* | *There is a neighbor of mine who normally delivers in the house… when I saw her in real stomachache (labor) I just called the motorbike man. And we forced her until she came to the hospital here… Now when her husband came and asked, I told him I put her in a motor bike and she went to the hospital and he told me, “but that woman normally performs delivery on her own. I told him that we were told that delivery is done at the hospital because delivery can become difficult. (First time Adolescent Mother, KE)*  *I have shared with many people especially about the umbilical cord, that when you deliver a baby you don’t place them in water immediately. And I saw, my neighbor delivered, and I went to her place after one day, I found she had put the baby in water … I told her that with this you can harm the baby … it can cause an infection. So, she left the baby for three days and just wiped it. (Woman, KE)*  *After getting the knowledge from the group, I had a friend who had delivered, and the baby was experiencing challenges, so I told her to stop feeding with new foods and practice breastfeeding up to six months. I emphasized to her that she should do exclusive breastfeeding so that she can avoid the problem of the baby while growing. (Woman, KE)* |
