# Supplementary material for: Transforming women’s and providers’ experience of care for improved outcomes: A theory of change for group antenatal care in Kenya and Nigeria
Source: PLoS One. 2022 May 3;17(5):e0265174. doi: 10.1371/journal.pone.0265174 (PMC9064109; doi:10.1371/journal.pone.0265174)
Supplement: S3 Table — (DOCX) [file pone.0265174.s006.docx]

**S3 Table: Topics discussed between group members outside of G-ANC meetings.**

|  | **Nigeria**  **N=405***  **N(%)** | **Kenya**  **N=280***  **N(%)** |
| --- | --- | --- |
| **Upcoming meetings** | 303 (74.8) | 155 (55.4) |
| **Problem someone having** | 42 (10.4) | 46 (16.4) |
| **Help someone needed** | 54 (13.3) | 26 (9.3) |
| **Advice/information related to:** |  |  |
| **Pregnancy** | 92 (22.7) | 79 (28.2) |
| **Family planning** | 79 (19.5) | 53 (18.9) |
| **Parenting** | 73 (18.0) | 73 (26.1) |
| **Infant/child care practices or health** | 109 (26.9) | 75 (26.8) |

Multiple choices allowed

*N=Women who attended at least one group meeting
